# Comparative Transcriptome Analysis of Two Olive Cultivars in Response to NaCl-Stress

**DOI:** 10.1371/journal.pone.0042931

**Published:** 2012-08-30

**Authors:** Christos Bazakos, Maria E. Manioudaki, Ioannis Therios, Demetrios Voyiatzis, Dimitris Kafetzopoulos, Tala Awada, Panagiotis Kalaitzis

**Affiliations:** 1 Department of Horticultural Genetics & Biotechnology, Mediterranean Agronomic Institute of Chania (MAICh), Crete, Greece; 2 Department of Horticulture, Aristotle University of Thessaloniki, Thessaloniki, Greece; 3 Institute of Molecular Biology and Biotechnology (IMBB), Foundation of Research and Technology (FORTH), Crete, Greece; 4 School of Natural Resources, University of Nebraska–Lincoln, Lincoln, Nebraska, United States of America; University of Toronto, Canada

## Abstract

**Background:**

Olive (*Olea europaea L.*) cultivation is rapidly expanding and low quality saline water is often used for irrigation. The molecular basis of salt tolerance in olive, though, has not yet been investigated at a system level. In this study a comparative transcriptomics approach was used as a tool to unravel gene regulatory networks underlying salinity response in olive trees by simulating as much as possible olive growing conditions in the field. Specifically, we investigated the genotype-dependent differences in the transcriptome response of two olive cultivars, a salt-tolerant and a salt-sensitive one.

**Methodology/Principal Findings:**

A 135-day long salinity experiment was conducted using one-year old trees exposed to NaCl stress for 90 days followed by 45 days of post-stress period during the summer. A cDNA library made of olive seedling mRNAs was sequenced and an olive microarray was constructed. Total RNA was extracted from root samples after 15, 45 and 90 days of NaCl-treatment as well as after 15 and 45 days of post-treatment period and used for microarray hybridizations. SAM analysis between the NaCl-stress and the post-stress time course resulted in the identification of 209 and 36 differentially expressed transcripts in the salt–tolerant and salt–sensitive cultivar, respectively. Hierarchical clustering revealed two major, distinct clusters for each cultivar. Despite the limited number of probe sets, transcriptional regulatory networks were constructed for both cultivars while several hierarchically-clustered interacting transcription factor regulators such as JERF and bZIP homologues were identified.

**Conclusions/Significance:**

A systems biology approach was used and differentially expressed transcripts as well as regulatory interactions were identified. The comparison of the interactions among transcription factors in olive with those reported for Arabidopsis might indicate similarities in the response of a tree species with Arabidopsis at the transcriptional level under salinity stress.

## Introduction

Olive (*Olea europaea L.*) is one of the most significant fruit-tree crop species of the Mediterranean region. Olive culture rapidly expands and trees are often irrigated with low quality, mostly saline water during the summer period. The concept of tolerance to salinity stress for most Mediterranean evergreen sclerophylls such as *Olea europaea L.* mostly refers to the ability of a plant to survive rather than to the reduction of its growth rate [1]–[4]. However, saline water negatively affects olive shoot growth [5], [6], causes morphological changes in leaves [7] and affects fruit productivity [8]. Moreover, it has been observed that the rate of photosynthesis decline [9], [10], the concentration of K^+^ is reduced due to the K^+^ - Na^+^ antagonism which impairs the function of K^+^ [11] while salt-induced osmotic adjustments take place as a result of compatible solutes accumulation [12], [13].

The tolerance of olives to salinity (NaCl) is believed to be intermediate [14]. However, there are salt-tolerant and salt-sensitive genotypes considering the extended genetic diversity of olive germplasm [15]. Tolerant genotypes have greater ability to exclude toxic ions and control the net salt import to the shoot [13] and therefore limit the accumulation of Na^+^ and Cl^−^ in actively growing shoots and leaves [16]. This strategy prevents salt translocation rather than salt absorption [17]–[19].

A common strategy for the identification of genes related to salt stress is the comparative study of relative species or cultivars with variation in the tolerance to this abiotic stress [20]. Such a strategy mainly relies on comparative transcriptome analysis [20] using microarrays. A plethora of comparisons between salt-sensitive and salt-tolerant cultivars of model and non-model plant species such as Arabidopsis [21]–[23], rice [24], tomato [25], poplar [26], potato [27] and sugarcane [28] have been reported up to now. These reports resulted in the identification of more than 30 families of transcription factors comprising DREB, CBF, MYB, bZIP and zing-finger families which are involved in abiotic stress including salinity [29], [30].

Although there are numerous studies on the response mechanism of olive trees to salinity, they are restricted to the ecophysiological level or to the study of a single pathway such as the mannitol metabolism in response to salt stress [31]. As a result, the molecular basis of salt tolerance in olive at a systems level has not been investigated yet. We initiated a comparative study to investigate the molecular response of a salt-sensitive and a salt-tolerant cultivar at the transcriptome level. The experiment was conducted using potted plants grown in ambient conditions, comprising of one-year-old olive trees treated with 120 mM NaCl for 90 days followed by a post-stress period of 45 days. This 135-day long study was conducted during the summer period when the needs for irrigation are maximized and the irrigation water is mostly saline.

Transcriptomic profiles for two *Olea europaea L.* cultivars under salinity conditions were generated and interacting relations among transcription factors and target transcripts were identified. We therefore hypothesized the transcriptional regulatory networks that are shaped during stress response through reverse-engineering of gene expression profiles [32], [33] using LeMoNe (Learning Module Networks) algorithm, a probabilistic module networks framework that has been applied on various other biological data sets [34]–[37].

## Results

### Olive Expressed Sequence Tag (EST) analysis and microarray construction

A complementary DNA (cDNA) library was constructed from total RNA extracted from two-month-old olive (cv. Koroneiki) seedlings and used for random single pass sequencing of 2643 cDNA clones resulting in 1956 ESTs with an average length of 381 bp (Figure S1). The sequences have been submitted to the EST database (dbEST, http://www.ncbi.nlm.nih.gov/projects/dbEST/) under accession numbers [dbEST: JK747885-JK749840]. SeqManII software (DNAstar Inc.) was used to cluster the cDNAs into 215 contigs and 996 singletons indicating that the redundancy of the EST collection (number of ESTs in clusters over total number of ESTs) was at the level of 49%. This relatively low redundancy was expected since most of the contigs (185 out of 215) comprised of a low number of ESTs (2–4) while only 14 contigs comprised more than 10 ESTs (Table S1).

**Figure 1:**
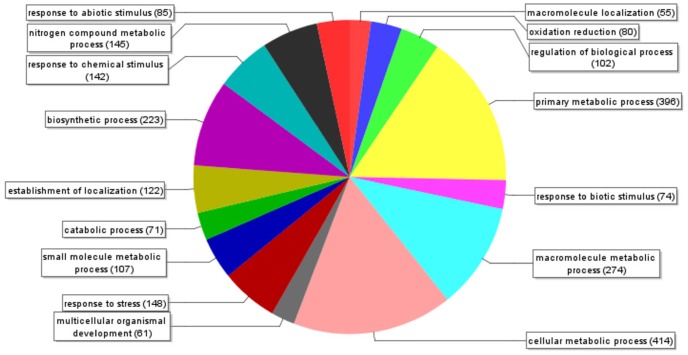
Pie chart of the EST annotation of olive seedling cDNA library. Biological process level 3 of 1956 transcripts according to Blast2GO database.

A blastx search of the 1211 non-redundant ESTs was carried out with an E-value of 1.0E-6 using Blast2GO [38]. A high percentage of the ESTs (33.5%) encoded for proteins with unknown function while 26.5% of ESTs showed no similarity to any sequence in public databases (Figure S2). Most of the annotated ESTs are related to primary, cellular, macromolecule and nitrogen compound metabolic processes (Figure 1). However, abiotic and biotic stress response-related ESTs represent the second most abundant group.

Following PCR amplification and purification of the above non-redundant ESTs, 200 custom aminosilane DNA microarrays were constructed containing 1121 cDNAs printed in triplicates. All microarray data from this study have been submitted to NCBI's Gene Expression Omnibus (http://www.ncbi.nlm.nih.gov/geo) under accession number [GEO: GSE36198]. The quality of the printed microarrays was confirmed (data not shown) prior to hybridization.

### Plant growth under NaCl stress

**Figure 2:**
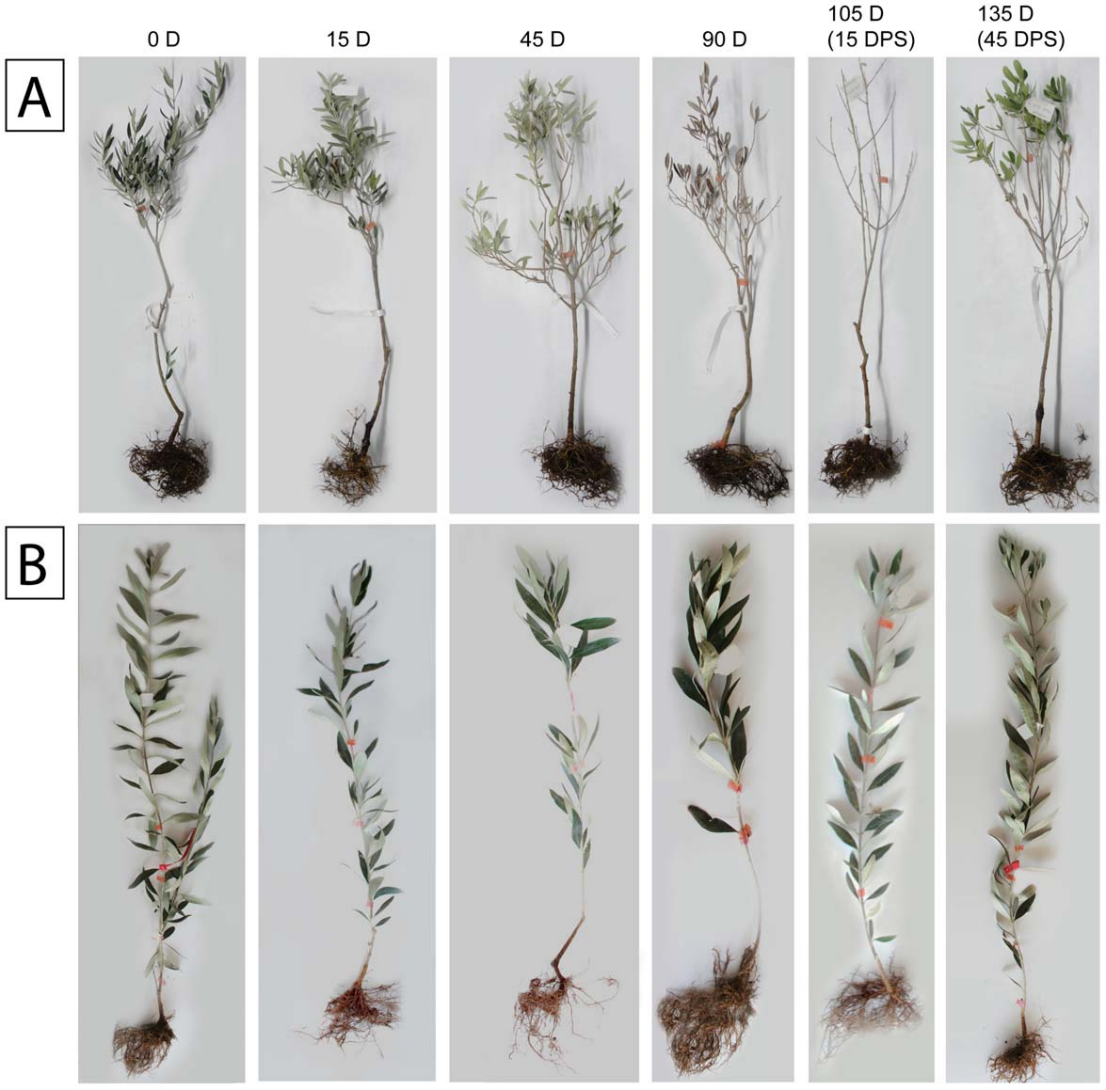
Visual symptoms of Na-Cl stress. One-year-old olive trees cvs Chondrolia Chalkidikis [A] and Kalamon [B] throughout the experimental timepoints: 15 days, 45 days and 90 days of stress and 15 days and 45 days post-stress.

One-year-old olive trees of cvs. Kalamon and Chondrolia Chalkidikis were treated with 120 mM NaCl under the ambient conditions of that particular summer period for a 90-day long time course followed by a post-stress period of 45 days. Visual symptoms of NaCl stress such as dead leaf edge, necrosis of stem tips and leaf drop appeared only in Chondrolia Chalkidikis, the sensitive cultivar, after 45 days, and became more severe after 90 days of treatment (Figure 2). No toxicity symptoms appeared in Kalamon, the tolerant cultivar, just a decrease in shoot growth after 45 days of treatment. Chondrolia Chalkidikis trees recovered within 45 days after the NaCl treatment (Figure 2).

### Significant Analysis of Microarrays (SAM) and K-means clustering

Root tissue was sampled after 15, 45 and 90 days of NaCl-treated and untreated olive trees as well as after 15 and 45 days of post-treatment period. Total RNA was extracted from the root samples of NaCl-treated and untreated olive trees and used for the microarray hybridizations. Microarray expression data for each time point per treatment were compared with each other in all possible combinations using a loop design (Figure S3) proposed by Yang and Speed [39] in an attempt to delineate the transcriptome response of the two cultivars under salinity. In total, 20 hybridizations per cultivar were performed within this experimental setup taking into account all possible combinations. Dye swap hybridizations and four biological replicates were performed for each time point and treatment.

We identified differentially expressed transcripts during the NaCl treatment as well as during the post-stress period. In total, 432 transcripts were used for subsequent analysis in Kalamon and 372 in Chondrolia Chalkidikis following the normalization procedure.

**Figure 3:**
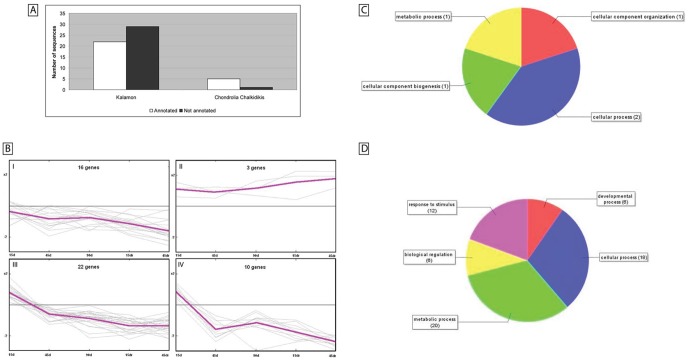
GO annotation of the differentially expressed transcripts. [A] 22/51 of the differentially expressed transcripts (2-class paired SAM) of cv. Kalamon and 5/6 of the differentially expressed transcripts (2-class paired SAM) transcripts of cv. Chondrolia Chalkidikis were annotated using the 1e-6 threshold of blastx in the Blast2GO software. [B] K-means clustering of the 51 Kalamon differentially expressed transcripts. Log_2_ (Treated/Control) gene expression data were best represented by four k-means clusters. Time and fold change are indicated on the x- and y-axes, respectively. In each cluster, the number of transcripts is indicated. A pseudoline in magenta colour superimposed on each cluster represents the general pattern of expression. [C] Pie chart of the cv. Chondrolia Chalkidikis annotated transcripts. [D] Pie chart of the cv. Kalamon annotated transcripts.

A two-class paired SAM was implemented using the MeV software [40] in order to compare gene expression between NaCl-treated and untreated root samples. In cv. Kalamon, 51 transcripts (11.8%) were found to be differentially expressed with a false discovery rate (FDR) of 4.93 (9.68%) and δ-value 0.81. Moreover, K-means clustering analysis revealed four distinct patterns of expression. One cluster represented gradually up-regulated transcripts, one down-regulated transcript while two clusters represented significantly suppressed transcripts in response to salinity (Figure 3B). The transcripts for each cluster as well as their corresponding annotation are listed in Table S2. Annotation of the differentially expressed transcripts showed that 22 out of the 51 are involved in the response to stimulus, biological regulation and developmental, cellular and biological processes. Only six (1.4%) transcripts were identified as differentially expressed in cv. Chondrolia Chalkidikis with an FDR rate of 1.5 (25%) and δ-value of 0.47. Gene Ontology (GO) annotation of the five cv. Chondrolia Chalkidikis transcripts showed involvement in cellular and metabolic processes (Figure 3C, D).

### Comparison between stress and post-stress conditions

Two-class unpaired SAM compared transcript expression data between the NaCl-stress and the post-NaCl stress time course resulting in the identification of 209 and 36 differentially expressed transcripts in cv. Kalamon and cv. Chondrolia Chalkidikis respectively, with a cut off FDR less than 6.5%.

**Figure 4:**
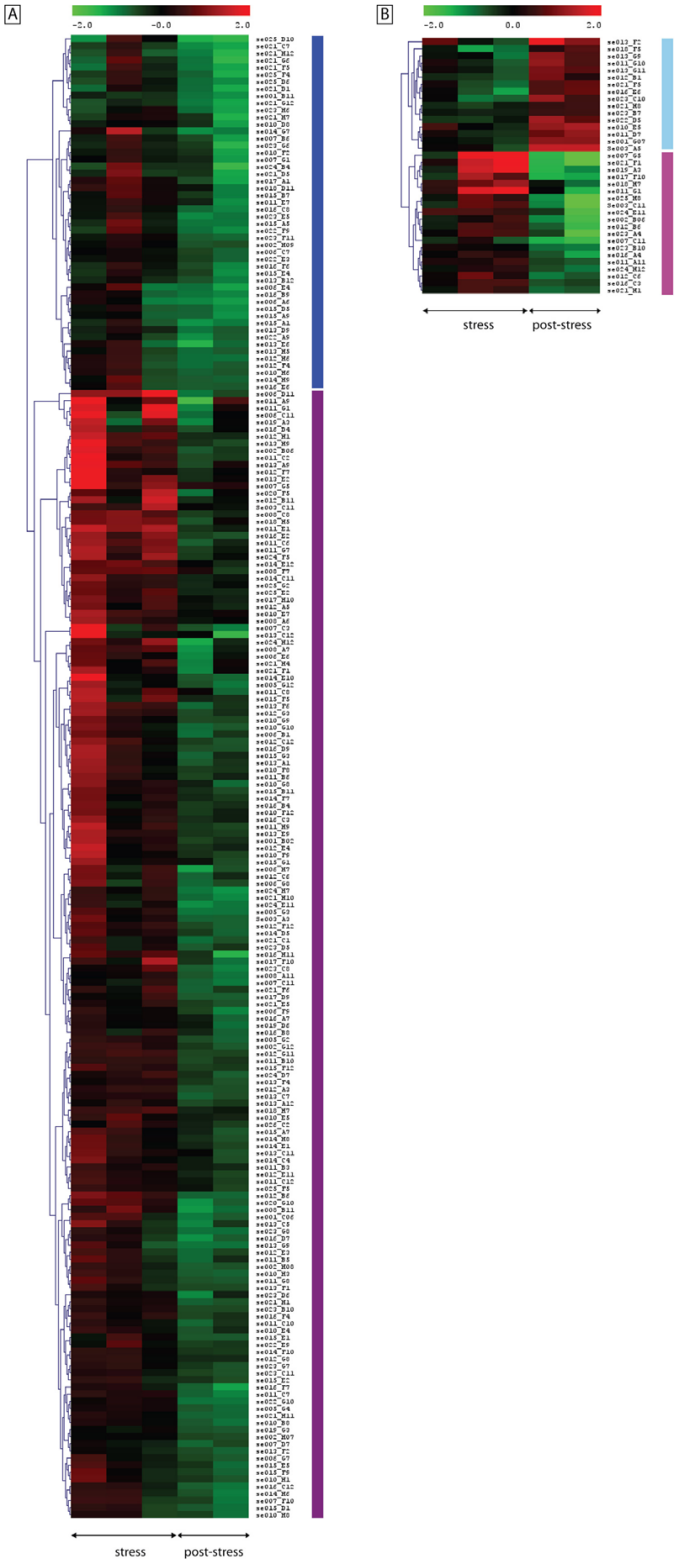
Differentially expressed transcripts between the stress and the post-stress period. Hierarchical clustering revealed the existence of distinct clusters in both cultivars. [A] In cv. Kalamon, 209 differentially expressed transcripts were hierarchically grouped in two major clusters denoted with two vertical bars. The blue bar represents a cluster comprised of 50 transcripts which are up-regulated only after 45 days of stress. The magenta bar represents a cluster comprised of 159 transcripts which are up-regulated throughout the stress period. [B] In cv. Chondrolia Chalkidikis, 36 differentially expressed transcripts were hierarchically grouped in two major clusters denoted with two vertical bars. The light blue bar represents a cluster of 16 transcripts which are down-regulated during stress and up-regulated during post-stress period. The light magenta bar represents a cluster comprised of 20 transcripts which are up-regulated after 45 and 90 days of stress and down-regulated during post-stress period.

Hierarchical clustering of these transcripts revealed two major clusters for each cultivar. The two clusters showed distinct patterns of expression in response to NaCl stress. In the first cluster of 50 transcripts in cv. Kalamon, transcript expression declined after 15 days of stress, peaked after 45 days and declined again by the end of the 90-day treatment. The second cluster of 159 transcripts showed exactly the opposite pattern of expression with up-regulation after 15 and 90 days and down-regulation after 45 days of stress. However, all transcripts were significantly down-regulated in the post-stress period (Figure 4).

In cv. Chondrolia Chalkidikis, the first cluster of 16 transcripts was down-regulated during the stress period but up-regulated during post-stress while the second cluster of 20 transcripts showed exactly the opposite pattern of expression with up-regulation during stress but down-regulation during post-stress.

**Figure 5:**
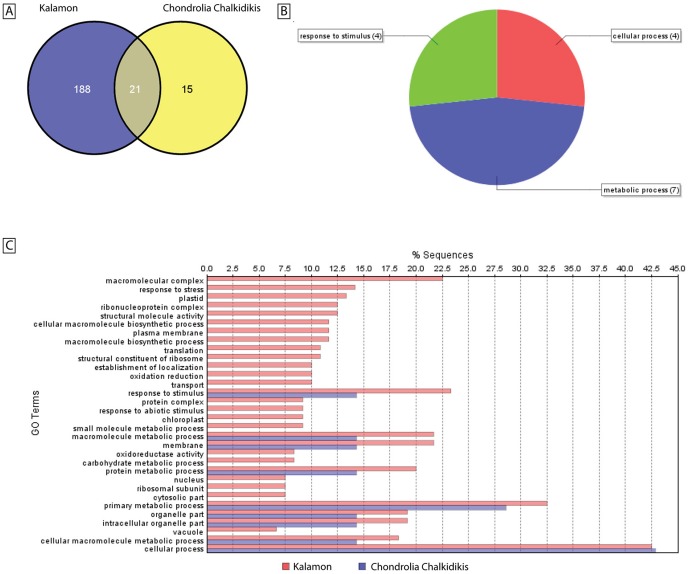
Comparison, GO annotation and Fisher's exact test of differentially expressed transcripts under stress and post-stress. [A] Venn diagrams of the differentially expressed transcripts in cross-cultivar microarray comparisons. The Venn diagrams show the number of overlapping and non-overlapping transcripts with significantly differential expression between stress and post-stress period. 188 unique transcripts were identified in cv. Kalamon and 15 in cv. Chondrolia Chalkidikis while 21 transcripts were common in both cultivars. [B] GO annotation level 2 of the 21 transcripts. [C] Fisher's exact test for the probability that the observed distribution of terms in the set of cv. Kalamon transcripts was significantly different (FDR<0.05) from the set of cv. Chondrolia Chalkidikis transcripts.

The two cultivars share 21 differentially expressed transcripts in response to NaCl-stress which are implicated in cellular and metabolic processes as well as in response to stimulus according to the Venn diagram (Figure 5A, B).

A two-tailed Fisher's exact test was performed in order to assess whether the frequency of functional categories comprising the transcripts of cv. Kalamon differ from those of cv. Chondrolia Chalkidikis. Several GO terms such as ‘cellular’, ‘primary metabolic’, ‘cellular macromolecule metabolic’ and ‘macromolecule metabolic processes’ were common in both cultivars as well as the ‘response to stimulus’ which is implicated in stress response. However, there are differentially expressed transcripts overrepresented in several GO terms involved in abiotic stress only in cv. Kalamon (Figure 5C).

### Transcriptional regulatory networks

Expression profiles can be used to infer regulatory networks and key transcription factors [41]. We constructed two regulatory module networks, one for each cultivar, using the LeMoNe algorithm [42] and our microarray data. The output of the algorithm was a set of modules of co-expressed transcripts, with a list of high-scoring transcription factor (TF) regulators attached to each cluster, prioritized according to their corresponding weight.

#### Kalamon regulatory network

**Figure 6:**
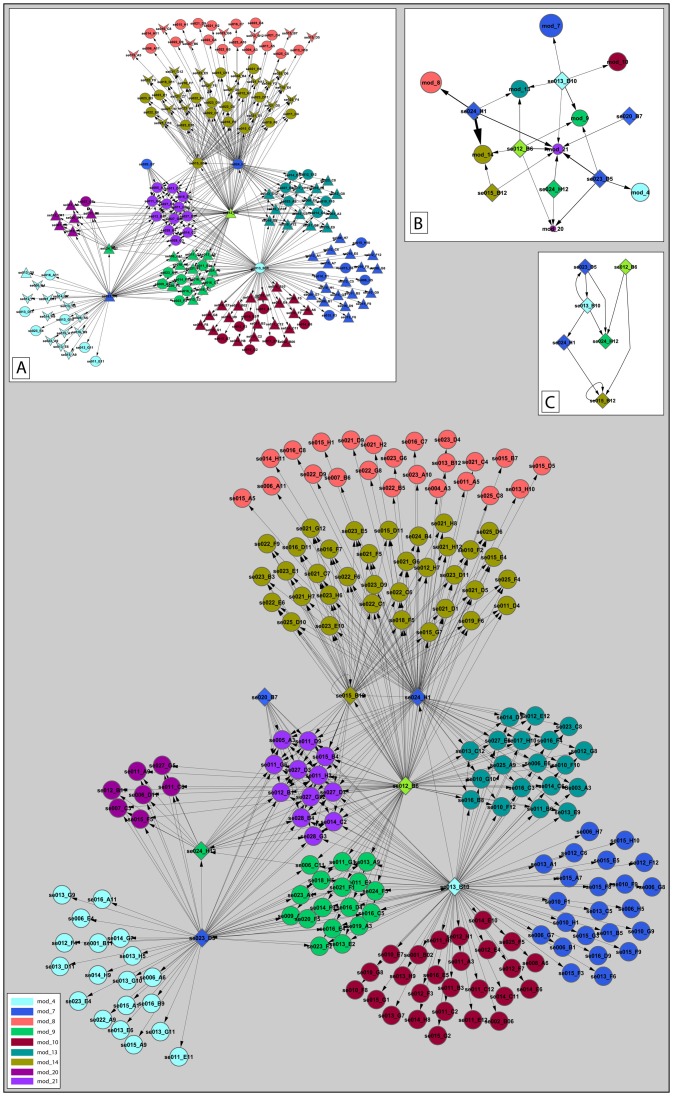
Transcriptional regulatory network of cv. Kalamon under salinity. A total number of 186 transcripts participated in the regulatory program, clustered in 9 non-overlapping modules (main frame). Modules are color-coded as indicated in the left-down inset. Diamonds represent transcripts encoding transcription factors (TFs) whereas circles represent transcripts regulated by TFs. The directionality of the regulation is indicated through arrowed edges heading from the TF to the target gene. [Inset A] Transcripts are divided into three categories. The triangles represent differentially expressed transcripts which are grouped in the magenta-colored cluster (see Figure 4A); V-shapes represent differentially expressed transcripts which are grouped in the blue-colored cluster (see Figure 4A). The circles represent non-differentially expressed transcripts. [Inset B] An abstract representation of the transcriptional regulatory network. The diamonds represent TFs. The circles represent modules and the length of their diameter is proportional to their transcript number. The color of each module is similar with those of the main frame. The width of the arrow is proportional to the weight of the regulatory interaction. [Inset C] The hierarchical TF regulatory network. The color of each TF is similar to the color of the module it belongs. The directionality of the regulation is indicated through arrowed edges.

In total, twenty-two modules were constructed for cv. Kalamon, each comprised of 7–36 transcripts (Figure S4). In addition, at least one high-scoring regulatory TF was identified for nine of these modules. The module network with the highest score comprised of 186 transcripts, clustered in nine non-overlapping modules and regulated by seven TFs (Figure 6). A simplified version of the module network (Figure 6B) suggests the coordinated effort of transcript expression regulation in the salt-tolerant cv. Kalamon. Moreover, six out of the seven TFs form a regulatory cascade. Specifically, three TF homologs, bZIP (se013_B10), NF-YC (se023_D5) and NF-YB (se012_B6) were placed upstream in the hierarchical network shown to regulate the action of three more TF homologs: JERF (se024_H1), HMG (se024_H12) and GRAS (se015_B12), considering that transcriptional regulation is a multi-layer hierarchical process [32] (Figure 6C).

JERF and bZIP homologs regulated module-13 that comprised differentially expressed transcripts which were up-regulated during stress and down-regulated during post-stress according to hierarchical clustering analysis. Transcripts encoding a nucleotide-sugar transporter (se023_C8), an ubiquitin conjugating enzyme (se012_G8) and an aldehyde dehydrogenase (se016_C3) were included in this module. The positive role of ubiquitin conjugating enzymes in drought and salt tolerance was demonstrated by over-expression studies in Arabidopsis [43].

#### Chondrolia Chalkidikis regulatory network

**Figure 7:**
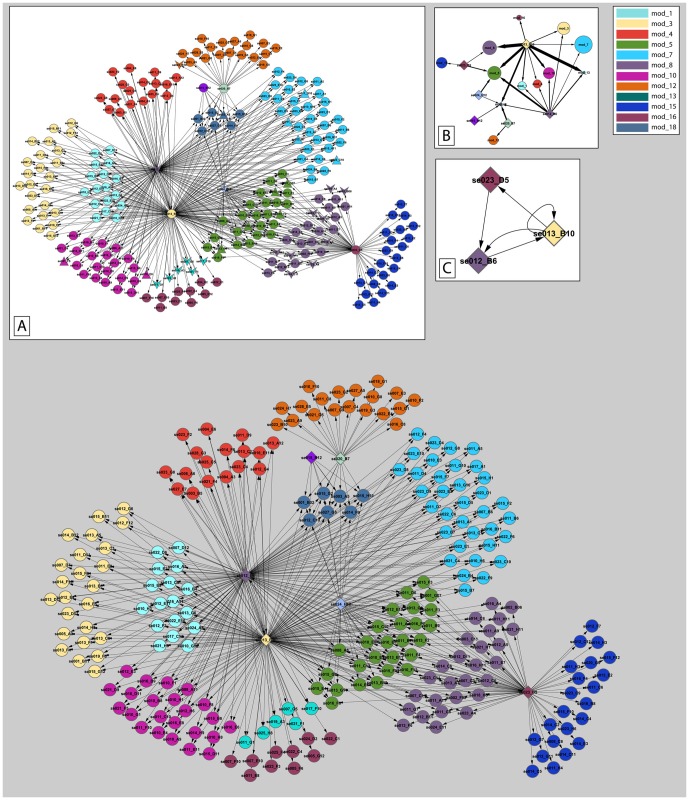
Transcriptional regulatory network of the response of cv. Chondrolia Chalkidikis under salinity. A total number of 237 transcripts participated in the regulatory program, clustered in 12 non-overlapping modules (main frame). Modules are color-coded as indicated in the right-up inset. Diamonds represent transcripts encoding transcription factors (TFs) whereas circles represent transcripts regulated by TFs. The directionality of the regulation is indicated through arrowed edges heading from the TF to the target gene. [Inset A] Transcripts are divided into three categories. The triangles represent differentially expressed transcripts which are grouped in the magenta-colored cluster (see Figure 4B); V-shapes represent differentially expressed transcripts which are grouped in the blue-colored cluster (see Figure 4B). The circles represent non-differentially expressed transcripts. [Inset B] An abstract representation of the transcriptional regulatory network. The diamonds represent TFs. The circles represent modules and the length of their diameter is proportional to their transcript number. The color of each module is similar with those of the main frame. The width of the arrow is proportional to the weight of the regulatory interaction. [Inset C] The hierarchical TF regulatory network. The color of each TF is similar to the color of the module it belongs to. The directionality of the regulation is indicated through arrowed edges.

Module network analysis of cv. Chondrolia Chalkidikis gene expression data revealed twenty modules comprised of 6–35 transcripts each (Figure S4). The module network with the highest score comprised of 237 transcripts, clustered in twelve non-overlapping modules and regulated by six TFs (Figure 7). Although the simplified version of the cv. Chondrolia Chalkidikis module network (Figure 7B) does not differ from cv. Kalamon's network in terms of complexity, the TF regulatory cascade of cv. Chondrolia Chalkidikis comprised of only three TFs which are mutually regulated (Figure 7C). This suggests a lower level of TF coordination in the salt-sensitive cv. Chondrolia Chalkidikis.

#### Incorporation of two-class unpaired SAM

The differentially expressed transcripts according to the two-class unpaired SAM between the NaCl-stress and the post-stress time course were mapped in the module networks of cv. Kalamon and cv. Chondrolia Chalkidikis (Figure 6A, 7A). In cv. Kalamon, eight out of nine modules consist of transcripts which are differentially expressed while seven out of eight contain at least 50% differentially expressed transcripts. Moreover, the pattern of differentially expressed transcripts is strongly module-dependent following the hierarchical clustering. Specifically, module-4, module-8 and module-14 are down-regulated while module-7, module-9, module-10, module-13 and module-20 are up-regulated during the stress period. However, in cv. Chondrolia Chalkidikis, only six out of twelve modules comprise transcripts which are differentially expressed, five of which comprise less than 35% differentially expressed transcripts.

### Enrichment of GO categories in modules

We calculated enrichment of Gene Ontology (GO) categories for every cluster in the two cultivars using the FatiGO tool [44] implemented in Blast2GO software [38]. A total of fifteen out of twenty two clusters in cv. Kalamon and sixteen out of twenty in cv. Chondrolia Chalkidikis have at least one GO category overrepresented at the 1.0E-3 significance level. There are 122 different GO categories overrepresented in cv. Kalamon (Table S3) and 115 in cv. Chondrolia Chalkidikis (Table S4). Several modules are enriched for stimulus-response categories in both cultivars, in particular module-2, module-7, module-9, module-10 and module-22 in cv. Kalamon and module-6, module-7, module-12 and module-20 in cv. Chondrolia Chalkidikis.

Module-9 in cv. Kalamon is enriched for response to stimulus and regulated by three high-scoring TF regulators. Blastx analysis of transcript se013_B10 showed 63% similarity to a bZIP TF. Members of the bZIP family have been reported to be responsive to salt stress in Arabidopsis [45], [46]. Transcript se012_B6 showed 96% similarity to a DR1-like protein that has previously been implicated in Arabidopsis stress response [47]. These two TFs regulated module-7 in cv. Chondrolia Chalkidikis which is enriched in stimulus-related GOs such as ‘cellular response to stimulus’ and ‘cellular response to organic substance’. The third regulator, transcript se023_D5, showed 95% similarity to a CCAAT-binding transcription factor. CCAAT elements are bound by nuclear factor Y (NF-Y) including a DR1 protein [48] which plays an important role in the endoplasmic reticulum-response in Arabidopsis [49] and oxidative stress response in eukaryotes [50].

Other modules are also statistically enriched for various stimulus functional categories like ‘ion binding’ (module-2), ‘response to ethylene stimulus’ (module-7), ‘response to temperature’ and ‘abiotic stimulus’ (module-10) and ‘response to fungus’ (module-22).

In cv. Chondrolia Chalkidikis, other modules with stimulus-related overrepresented GOs included module-6 (‘response to herbicide and toxin’), module-12 (‘response to cadmium’, ‘response to metal ion’ and ‘response to inorganic substance’) and module-20 (‘response to copper ion’).

### Comparison of Kalamon and Chondrolia Chalkidikis networks

**Figure 8:**
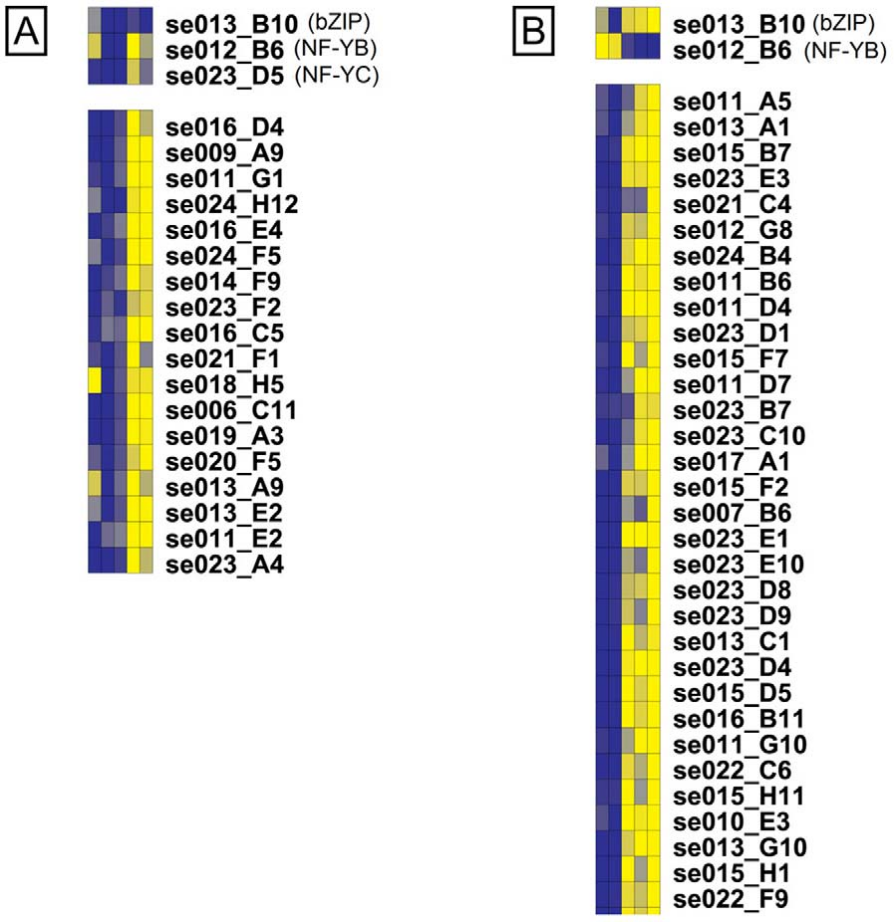
Expression panel of selected regulatory networks. The upper panel represents the regulatory TFs of a module and the lower panel the transcripts it consists of. Each column represents a time-point. The expression of the transcripts and TFs is color-coded with the dark blue representing lower expression whereas the bright yellow represents higher expression. [A] module-9 in cv. Kalamon; [B] module-7 in cv. Chondrolia Chalkidikis. Both modules are enriched in GO annotations related to stimulus response.

Two TFs, bZIP (se013_B10) and NF-YB (se012_B6) homologs, regulated modules in both cultivars related to stimulus response: module-7 in cv. Chondrolia Chalkidikis and module-9 in cv. Kalamon [45]–[49]. In addition, a third TF, NF-YC (se023_D5), appeared also as a co-regulator for stress-related module-9 in cv. Kalamon. However, it is interesting to note that different transcripts are regulated by the same olive bZIP and NF-YB homologues in the two cultivars (Figure 8). This might be explained by the opposite patterns of expression of both TFs in the two cultivars during the stress and post-stress period.

The Kalamon and Chondrolia Chalkidikis module networks shared 69 interactions which were mediated by three TFs. Some of these common interactions comprise differentially expressed transcripts such as an auxin-repressed protein (se002_B6), a catalase (se019_A3) and a major latex protein (se021_F1) known to be involved in stress responses [25], [51]. In addition, a putative SUMO (Small Ubiquitine-like MOdifier) protease homologue is regulated by bZIP in both cultivars but it is differentially expressed only in cv. Kalamon. SUMO proteases are considered regulators of signalling proteins in eukaryotes through SUMOylation [52] which plays an important role in abiotic stress, ABA signalling, pathogen defense and flowering time [53].

**Figure 9:**
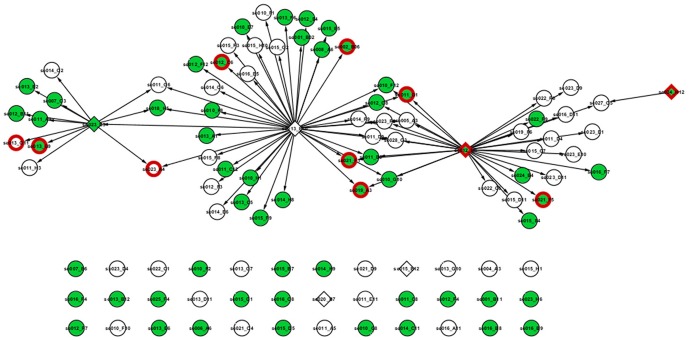
Intersection of the cv. Kalamon and cv. Chondrolia Chalkidikis transcriptional regulatory networks. 69 nodes share an edge that corresponds to a regulatory interaction between a TF and a transcript while 36 nodes are isolated, implicating their presence in both networks but with different interacting pairs. Diamond-shaped nodes represent TFs, circles represent regulatory transcripts. Green nodes are differentially expressed transcripts in cv Kalamon and red nodes are differentially expressed transcripts in cv Chondrolia Chalkidikis.

The intersection network (Figure 9) also contains 36 isolated nodes which represent transcripts that are present in both networks but with different interacting pairs. Among these are the CCR4-associated factor 1 (CAF1) (se020_B7) and GRAS (se015_B12) transcripts which are both related to stimulus-response terms according to Blast2GO annotation.

## Discussion

We conducted a 135-day long comparative salinity experiment with two olive cultivars, one sensitive and one tolerant to salinity. One-year-old trees were exposed to NaCl stress for 90 days followed by 45 days of post-stress period during the summer. Our objective was to mimic olive growing field conditions considering that experimental conditions greatly affect transcriptome responses in abiotic stress studies [20]. Therefore, we took into consideration the concentration of NaCl treatment by using 120 mM NaCl which olive trees possibly encounter when irrigated with low quality saline water, as well as the kinetics of stress treatment by implementing a long exposure to salinity covering the entire summer period. This way, the shortcomings of commonly used NaCl-stress in plastic jars and petri dishes and the rapid NaCl shock [54] were avoided.

Recent efforts were also reported in olive EST identification mainly related to pollen allergens and olive fruit ripening [55]. In this study entire olive seedlings of one month old were used to extract RNA for the cDNA library construction considering that the transcriptome of actively growing seedlings will be highly enriched with transcripts involved in growth and development. This way, the enrichment of the cDNA library with transcripts involved in root and leaf growth programmes was much higher.

This cDNA library was sequenced and an olive microarray was constructed comprising 1121 non-redundant ESTs. Despite the limited probe set due to the low number of ESTs comprising the olive microarray, transcriptional regulatory networks were constructed for a salt-tolerant and a salt-sensitive cultivar after transcriptome analysis. Moreover, several hierarchically-clustered interacting TF regulators were identified known to be involved in stress-adaptive responses including salinity.

In cv. Kalamon, 159 out of 209 transcripts were up-regulated throughout stress, while the remaining 50 were up-regulated only after 45 days of stress according to hierarchical clustering. All 209 transcripts were subsequently down-regulated during post-stress. However, a small number of 20 transcripts were up-regulated throughout stress in cv. Chondrolia Chalkidikis, a 10-fold decrease in transcript number, indicating limited transcriptional activation in the salt-sensitive cultivar. This lack of transcriptional activation might be partly responsible for the sensitivity of cv. Chondrolia Chalkidikis compared to cv. Kalamon at the gene expression level. Moreover, the 209 transcripts are characterized by GO annotations related to stress response, transport and response to abiotic stimulus which are not represented in the 36 differentially expressed transcripts of cv. Chondrolia Chalkidikis according to Fischer's exact test analysis. These GO annotations are directly involved in salinity stress confirming the lack of response of the salt-sensitive cultivar at the transcriptional level.

Within the 209 differentially expressed transcripts in cv. Kalamon there are seven which might be considered salt-specific such as a salt-stress responsive-encoding putative small glutamine rich tetratricopeptide repeat containing protein, 40 s ribosomal protein, NAD+ ADP-ribosyltransferase, annexin A4, xyloglucan endotransglycosylase, UDP-galactose epimerase and stress-induced protein.

Although in cv. Kalamon most of the transcripts were down-regulated in the post-stress period, in cv. Chondrolia Chalkidikis a cluster of 16 transcripts was clearly up-regulated during post-stress. GO annotation of these transcripts indicated their involvement in metabolic, cellular and developmental processes presumably in order to grow new leaves since the old ones senesced and abscised after 90 days of salinity. Such an example is a putative serine c-palmitoyltransferase-like protein homologue, which is known to be involved in cell growth according to GO annotation and is upregulated during post-stress only in cv. Chondrolia Chalkidikis but not in cv. Kalamon.

Regulatory networks were constructed in the context of adaptive mechanisms to salinity stress considering the lack of previously identified networks of this kind in any tree species and the assumption that the efficiency of plant adaptation to salinity would probably increase through modulation of salt-responsive gene regulatory networks. This strategy would require the initial characterization and the subsequent alteration of expression of key transcription factors in order to enhance salt stress tolerance [56].

To this direction, we characterized several olive transcription factor homologs. The olive JERF homolog belongs to the family of ethylene response factors (ERFs). The JERF TFs are known to be involved in abiotic stress responses including salinity through regulation of expression of stress-responsive and ABA biosynthesis-related genes [57]. Although the JERF is up-regulated after 45 days of salinity in cv. Kalamon (data not shown) it is not expressed at all either during the stress or the post-stress period in cv. Chondrolia Chalkidikis indicating a significant difference in regulation of a stress-responsive transcription factor between the salt-sensitive and salt-tolerant cultivar. Therefore, the JERF homolog might play a key role in olive adaptation to salinity. The pattern of expression of the JERF homolog should be determined under salinity in additional salt–sensitive and salt–tolerant genotypes in order to assess its potential to be used as a molecular marker of salt tolerance.

In addition, the olive bZIP homolog seems to play a key role in the highly complex gene regulatory network in cv. Kalamon as well as in cv. Chondrolia Chalkidikis through regulation of other TFs, members of the module network. The expression of the putative olive bZIP homolog was up-regulated in cv. Chondrolia Chalkidikis, the salt-sensitive cultivar, while it remained steady in cv. Kalamon, the salt-tolerant cultivar, after 15 and 45 days of treatment (Figure 8). These patterns of olive bZIP expression are in agreement with the patterns of Arabidopsis bZIP24 expression and its salt-tolerant relative species *Lobularia maritime*. In Arabidopsis, a salt-sensitive species, the bZIP24 with homology to olive bZIP was up-regulated, while in *Lobularia maritime* it was repressed [58]. Moreover, the olive bZIP regulated module networks comprising transcripts induced in cv. Kalamon but suppressed in cv. Chondrolia Chalkidikis in response to salt. This is in agreement with the RNAi-mediated repression of bZIP24 in Arabidopsis which increased stress tolerance due to the transcriptional activation of a wide range of stress-induced genes involved in cytoplasmic ion homeostasis, osmotic adjustment and plant growth and development [58]. The AREB1, a group A bZIP, was also shown to play a key role in ABA signalling under drought stress [59].

Moreover, the hierarchical relation between the olive bZIP and the olive JERF homologs is identical to the one proposed by Golldack et al. [56] with the bZIP regulating JERF indicating similarities in transcription factor networks between Arabidopsis and a tree species such as olive.

In addition, two NF-Y TFs were placed upstream hierarchically in the regulatory network, indicating a key role in salt stress response of olive (Figure 6C). This is in agreement with the improved drought tolerance under field conditions exhibited by transgenic maize plants over-expressing the ZmNF-YB2 TF [60].

The olive bZIP homolog regulated on its own or in combination with other TFs five out of the nine modules in cv. Kalamon. Among the five modules, there are four which comprised differentially expressed transcripts indicating the central role of bZIP in salt response. One of these modules, module-13, is regulated by the bZIP and JERF homologs suggesting synergistic function. Moreover, the bZIP played a central role in cv. Chondrolia Chalkidikis's salinity response by regulating ten out of twelve modules.

The higher complexity of the cv. Kalamon transcription factor network compared to the cv. Chondrolia Chalkidikis network might be indicative of a more coordinated effort to adapt to salinity although a more representative number of transcripts have to be analyzed to draw reliable conclusions on olive response to salt.

The comparison of the salt-responsive transcriptional regulatory networks in olive with those reported for Arabidopsis [56] suggests that the regulons in both species might be comprised of some similar regulatory TF homologues. This indicates that a tree species might respond in a similar way to Arabidopsis at the transcriptome level under salinity stress. However, further research is required on olive transcriptome analysis to further support this assumption.

## Materials and Methods

### Plant material and salinity treatment

One-year-old, self-rooted trees of *Olea europaea* L., cvs. Kalamon and Chondrolia Chalkidikis, were grown under identical conditions by the commercial nursery “Kostelenos”, Poros-Trizinia, Greece, transported to Chania, Greece and transplanted in 4-lt plastic bags containing a 1∶1 mixture of perlite∶sand. The young trees were irrigated daily with 150 ml of half-strength Hoagland solution [61] for one month and 35 of those of similar growth were selected for the salinity experiment while another 35 for the un-treated control. In total, 70 young trees per cultivar were used for the experiment. The half-strength Hoagland solution [61] was supplemented with 120 mM NaCl for the salinity treatment. The experiment was conducted at the ambient temperatures of this particular summer at the premises of the Mediterranean Agronomic Institute at Chania, Crete, Greece and not in a growth chamber under controlled environmental conditions. It started on the 15th of May and ended 135 days later throughout the entire summer period.

### RNA extraction and cDNA library construction

Four young trees were used to collect root tissue at each time point after 15, 45 and 90 days of treatment. The root tissue was then washed repeatedly with deionised water, sterilized with 0.5% sodium hypochloride, immediately ground with liquid nitrogen and stored at a −80°C freezer. Total RNA was extracted according to the method of Bachem et al. (1998) and concentrated using the NucleoSpin® RNA Clean-up XS kit (Macherey-Nagel, Duren, Germany) in order to be used for the microarray analysis.

Total RNA was extracted from one-month-old olive seedlings and used for the construction of a cDNA library with the Creator SMART cDNA library construction kit (BD Bioscience-Clontech, Mountain View, Canada). cDNA fragments longer than 600 bp were selected and directionally ligated into the pDNR-lib vector (BD Clontech, California) at the SfiΙ restriction site. Plasmids were transformed into the *E. coli* strain DH10B (Invitrogen, Carlsbad, CA, USA) by electroporation. The presence and the length of inserts were tested by PCR using the primers pDNR-F_1∶5′- TAAAACGACGGCCAGTA-3′ and pDNR-R_1: 5′- GAAACAGCTATGACCA TGTTC-3′.

The cDNA inserts were sequenced in one direction with the pDNR forward (5′-TAAAACGACGGCCAGTA-3′) primer using Big Dye 3.1 chemistry on an ABI 3700 sequencer (Applied Biosystems, Foster City, CA, USA). The raw sequence reads were quality-trimmed and vector- and poly-A clipped. Clustering of the sequences was performed with the SeqManII software (DNASTAR, Inc. Madison, WI, USA) resulting in contigs and singletons.

### Homology search and GO annotation

GO terms were assigned after blastx search of 2600 unique EST sequences using Blast2GO [38]. Threshold cut-off was at E-value 1.0e-6 and the alignment length of 33 amino acids.

### Microarray production

The 1121 unique cDNAs were PCR-amplified using Taq polymerase (Minotech, Heraklion, Greece) with the primers (pDNR-F_2: 5′-CCGCATAACTTCGTATAGC–3′ and pDNR-R_2: 5′-CATGTTCACTTACCT ACTGG-3′) positioned by the polylinker site. The PCR products were purified using Nucleofast 96 columns (Macherey-Nagel, Duren, Germany). Approximately 3 µg of purified PCR product was placed in 384-well printing plates (Genetix, Hampshire, UK), dried at room temperature and resuspended in printing buffer containing 450 mM NaCl, 45 mM phosphate pH 7.0, 5% [v/v] formamide and 0.01% [v/v] maltoside at a final concentration of 200 µg/µl. Printing was performed in triplicates on home-made aminosilane slides using a Packard SpotArray24 spotter with four print-tips in the Post-Genomics Facility of IMBB-FORTH, Heraklion, Greece.

### RNA labelling and microarray hybridization and washing

Approximately 15 µg of total RNA were labeled with the SuperScript™ Plus Direct cDNA Labeling System (Invitrogen, Carlsbad, CA, USA) using Alexa Fluor®-labelled nucleotides. The reverse transcription reaction was performed in a 30-µL volume containing 15 µg total RNA with 5 µg of oligo(dT) primer, 10 mM dithiothreitol, 1× Nucleotide mix with Alexa Fluor®-555-aha-dUTP or Alexa Fluor®-647-aha-dUTP, 40 units RNaseOUT™ and 800 units of SuperScript III reverse transcriptase in 1× Superscript first-strand buffer. After incubation at 46°C for 3 hours, the reaction products were treated with 15 µL of 0,1 M NaOH, incubated at 70°C for 30 min and treated again with 15 µL of 0,1 M HCl for 3 minutes. Two samples were combined and purified using the PureLink™ PCR purification system (Invitrogen, Carlsbad, CA, USA). The only modification was the use of 20 µl low-temp hybridization buffer (Arrayit Co., Sunnyvale, CA, USA) as elution buffer. The probe was supplemented with 3.5 µl of 5 mg/ml salmon sperm DNA and 2 µl of yeast tRNA. The mixture was heated at 60°C for 5 minutes and was kept at 45°C before hybridization.

The probes were placed onto the array; the slides in a sealed hybridization cassette (Genomic Solutions Inc. Ann Arbor, MI, USA) and submerged in a 43°C water bath for 18 hours. The slides were then placed in washing solution 1 (1× SSC, 0.03% SDS) and then transferred to washing solution 2 (0.2× SSC) for 5 min with agitation followed by washing solution 3 (0.05× SSC), agitated gently for 5 min, spun at 750 rpm for 4 min and dried. Microarrays were scanned with a microarray scanner (model GenePix 4000B, Axon).

### Microarray experimental design

Microarray gene expression data for salt-treated and untreated olive roots were obtained by comparing directly the results of each treatment/timepoint with each other in all possible combinations using a loop design proposed by Yang and Speed [39] (Figure S3). Taking into account all possible combinations, 20 hybridizations per cultivar were performed in total within this experimental setup. Dye swap hybridizations and 4 biological replicates were performed for each treatment/timepoint.

### Microarray image analysis, data acquisition and normalization

The .tiff images were analyzed using GenePix Pro 6.0 software and spots were flagged by GenePix as ‘not found’ or ‘absent’. In addition, data spots in each array were also manually estimated for spot quality and were flagged as ‘bad’ (non-uniform, saturated or high background) or ‘absent’ (below background spots). The default local background subtraction method was used. The GenePix Results files (.gpr) were subsequently converted to TIGR MultiExperiment Viewer (.mev) files using the ExpressConverter v2.1 tool implemented in the TM4 microarray software suite [40]. ExpressConverter provides the Integrated Intensity Value (IIV) for each spot, that is the total measured intensity across the biological sample printed on a spot [62]. These values were then used as input for MIDAS v2.21.

The MIDAS normalization and filtering pipeline that we used includes the following steps in order: (1) locally weighted scatterplot smoothing regression (LOWESS): normalization in block mode was used to compensate for non-linear dye bias, (2) in-slide replicate analysis to normalize for the three in-slide replicates of each unique probe and (3) low-intensity filtering that removes intensity values<10000 in order to avoid spurious signal-intensity measurements. Low-quality array elements were eliminated by applying several filtering methods such as background checking for both channels with a signal/noise threshold of 2.0 and usage of flags for both channels. One bad tolerance policy parameter was set to generous. We evaluated the effect of the normalization pipeline by creating Ratio Intensity (RI) plots (data not shown) at the end of the normalization procedure, as described by Quackenbush [63].

### Microarray data analysis

Two-class paired Significance Analysis of Microarrays (SAM) [64] implemented in TIGR MEV 4.5.1 [40] was used for the identification of differentially expressed transcripts. The paired inputs for SAM analysis were the normalized intensity values of the control (C) and treatment conditions (T) for each treatment time-point (T_15d vs C_15d, T_45d vs C_45d, T_90d vs C_90d, T_15dr vs C_15dr and T_45dr vs C_45dr). Features with missing values for any of the conditions were excluded from the SAM analysis. The significance cutoff, the δ value, was adjusted (δ = 0.81 for cv. Kalamon and δ = 0.47 for cv. Chondrolia Chalkidikis) so as to set the median false discovery rate FDR<5. Differentially expressed transcripts were further analyzed using the K-means clustering algorithm implemented in TIGR MEV 4.5.1 [40]. The default Euclidean distance was used as a metric and the maximum number of iterations was set to 50.

To assess the differences in response between stress and recovery conditions, two-class unpaired SAM [64] implemented in TIGR MEV 4.5.1 [40] was used. The inputs for SAM analysis were the normalized log_2_ (Treated/Control) intensity values of the stress vs. recovery time-points. Features with missing values for any of the conditions were excluded from the SAM analysis. The significance cutoff, the δ value, was adjusted resulting in a median false discovery rate (FDR) of <6.5%. For all analyses, we performed 100 random permutations of the data to select the differentially expressed transcripts and were required to select S0 based on the default method of Tusher et. al. [64]. Differentially expressed transcripts were further analyzed using the Hierarchical Clustering algorithm implemented in TIGR MEV 4.5.1 [40] using the Euclidean metric and complete linkage clustering algorithm.

### Module networks

In order to infer the module networks for both cultivars the LeMoNe algorithm [37], [42] was used. LeMoNe uses ensemble-based probabilistic optimization techniques to identify clusters of co-expressed transcripts as well as their regulators [65]. First it searches for clusters of co-expressed transcripts and subsequently defines a regulatory program for each cluster. Local optima traps in the first step are avoided using a Gibbs sampling approach for two-way clustering of both transcripts and conditions [37]. The algorithm receives as input the expression profiles of transcripts across the experimental conditions as well as a list of potential regulators.

In this study the normalized log_2_ (Treatment/Control) values of both cultivars were used as transcript expression input. A list of 30 potential regulators was identified using GO annotation description as indicated by the terms ‘transcription factor activity’, ‘nucleic acid binding’, ‘DNA binding’, ‘regulation of transcription’, ‘transcription regulator activity’ and ‘transcription activator activity’ using Blast2GO tool. For each cultivar 10 independent Gibbs sampler runs were performed, initiated with 216 and 186 clusters for Kalamon and Chondrolia Chalkidikis respectively, representing the half number of transcripts for each strain. LeMoNe assigned the corresponding regulators in each cluster characterized by a particular weight. The final set of regulators was set to be those that exceed the threshold of 10% of the maximum weight for each strain.

The final output of the algorithm is a group of clusters composed of mutually exclusive co-expressed transcripts, with a list of high-scoring regulators attached to each cluster, prioritized according to the corresponding weight.

### GO annotation, functional analysis of clusters and Fisher's exact test

Transcripts were annotated using the publicly available Blast2GO package v.2.4.4 [38]. Initially a blastx was run though QBlast against the NCBI nr-database of 2010-07-09 using default parameter values. Following the mapping step, the annotation step was performed using an e-value threshold of 1.0e-6. A comparison against the InterPro domain database was used to increase the number of annotated sequences, using InterProScan [66]. Enrichment analysis was completed for every cluster in the two cultivars using the FatiGO tool [44] implemented in Blast2GO [38] at the 0.01 significance level. Enrichment analysis between Kalamon and Chondrolia Chalkidikis annotated sequences was performed using Fisher's exact test implemented in Blast2GO [38].

### Network visualization

Visualization of the regulatory networks was performed with Cytoscape [67] v. 2.7.0. Intersection network was constructed using the NetworkAnalyzer Cytoscape plugin [68].

## Supporting Information

Figure S1 Distribution of sequence length for the 1956 ESTs.
(TIF)

Figure S2 Distributions of the blastx analysis of the 1211 non-redundant ESTs. NoBlastHits: sequences returning no blast hits, NoMapping: Mapping step returned no results for the sequence, NoAnnot: Annotation step returned no results for the sequence, Annot: Sequences with a GO assignment, Total: total number of sequences.
(TIF)

Figure S3 Loop design of the time course experiment. C: Control; T: NaCl-treatement; 1: 15 days stress; 2: 45 days stress; 3: 90 days stress; 4: 15 days post-stress; 5: 45 days post-stress. Each arrow represents a two-dye hybridization pair among the experimental treatments. Every time point sample was hybridized four times, each time with RNA extracted from a different plant. The head and tail of each arrow represent the Alexa 647 and Alexa 555 dye respectively.
(TIF)

Figure S4 Transcript distribution in regulatory modules for cv. Kalamon and cv. Chondrolia Chalkidikis. The Kalamon network consists of 22 modules (white bars) comprising 432 transcripts. Nine of the modules marked with a star comprise 186 transcripts and are regulated by at least one transcription factor. The Chondrolia Chalkidikis network consists of 20 modules (stripped bars) comprising 372 transcripts. Twelve of the modules marked with a star comprise 237 transcripts and are regulated by at least one transcription factor.
(TIF)

Table S1 Redundancy level of the olive seedling cDNA library. The first column shows the number of ESTs that comprise a contig while the second column shows the total number of contigs with the corresponding number of ESTs.
(DOCX)

Table S2 GO categories for clusters of the differentially expressed transcripts in cv. Kalamon. Transcript name: The name of the transcript in a cluster. Hit description: The description of the GO term assigned to the transcript. GO-ID: The GO-ID term assigned to the transcript. Term: The GO term assigned to the transcript.
(DOCX)

Table S3 GO categories for modules that comprise cv. Kalamon transcriptional regulatory network. The first column shows the module number, the second column shows the number of transcripts that comprise the module. Columns three and four show the GO ID and GO Term respectively while the last column shows the p-value of the GO term assignment as calculated in the FatiGO tool.
(DOCX)

Table S4 GO categories for modules that comprise cv. Chondrolia Chalkidikis transcriptional regulatory network. The first column shows the module number, the second column shows the number of transcripts that comprise the module. Columns three and four show the GO ID and GO Term respectively while the last column shows the *p*-value of the GO term assignment as calculated in the FatiGO tool.
(DOCX)

## References

[1] Munns R (2002) Comparative physiology of salt and water stress. Plant, cell & environment 25: 239–250. 10.1046/j.0016-8025.2001.00808.x 11841667

[2] Tattini M, Montagni G, Traversi ML (2002) Gas exchange, water relations and osmotic adjustment in Phillyrea latifolia grown at various salinity concentrations. Tree physiology 22: 403–412. 11960765 10.1093/treephys/22.6.403

[3] Munns R (2005) Genes and salt tolerance: bringing them together. The New phytologist 167: 645–663. 16101905 10.1111/j.1469-8137.2005.01487.x

[4] Tattini M, Traversi M (2009) On the mechanism of salt tolerance in olive (Olea europaea L.) under low- or high-Ca2+ supply. Environmental and Experimental Botany 65: 72–81.

[5] Tattini M, Bertoni P, Caselli S (1992) Genotypic responses of olive plants to sodium chloride. Journal of Plant Nutrition 15: 1467–1485.

[6] Klein I, Ben-Tal Y, Lavee S, De Malach Y, David I (1994) Saline irrigation of cv. Manzanillo and Uovo di Piccione trees. Acta Hort (ISHS) 356: 176–180.

[7] Therios IN (2009) Olives. CABI, U.K. p.

[8] Cresti M, Ciampolini F, Tattini M, Cimato A (1994) Effect of salinity on productivity and oil quality of olive (Olea europaea L.) plants. Advances in Horticultural Science 8: 211–214.

[9] Chartzoulakis K, Loupassaki M, Bertaki M, Androulakis I (2002) Effects of NaCl salinity on growth, ion content and CO 2 assimilation rate of six olive cultivars. Scientia Horticulturae 96: 235–247.

[10] Loreto F, Centritto M, Chartzoulakis K (2003) Photosynthetic limitations in olive cultivars with different. Environment 595–601.

[11] Therios IN, Karagiannidis N (1991) Effect of NaCl on growth and chemical composition of four olive cultivars (in Greek). Scientific Annals, School of Agriculture, Aristotle University of Thessaloniki. pp. 29–47.

[12] Tattini M, Gucci R, Romani A, Baidi A, John D (1996) Changes in non-structural carbohydrates in olive (Olea europaea) leaves during root zone salinity stress. 117–124.

[13] Gucci R, Lombardini L, Tattini M (1997) Analysis of leaf water relations in leaves of two olive (Olea europaea) cultivars differing in tolerance to salinity. Tree physiology 17: 13–21. 14759909 10.1093/treephys/17.1.13

[14] Rugini E, Fedeli E (1990) Olive (Olea europaea L) as an oilseed crop. Bajaj, Y.P. Berlin: Springer. p.

[15] Chartzoulakis K (2005) Salinity and olive: Growth, salt tolerance, photosynthesis and yield. Agricultural Water Management 78: 108–121.

[16] Melgar JC, Mohamed Y, Serrano N, García-Galavís PA, Navarro C, et al (2009) Long term responses of olive trees to salinity. Agricultural Water Management 96: 1105–1113.

[17] Benlloch M, Arboleda F, Barranco D (1991) Response of Young Olive Trees to Sodium and Boron Excess in Irrigation Water. Boron 26: 867–870.

[18] Tattini M, Gucci R, Coradeschi MA, Ponzio C, Everard JD (1995) Growth, gas exchange and ion content in Olea europaea plants during salinity stress and subsequent relief. Physiologia Plantarum 95: 203–210.

[19] Demiral MA (2005) Comparative response of two olive (Olea europaea L.) cultivars to salinity. Turkish Journal of Agriculture and Forestry 29: 267–274.

[20] Deyholos MK (2010) Making the most of drought and salinity transcriptomics. Plant, cell & environment 33: 648–654. 10.1111/j.1365-3040.2009.02092.x 20002333

[21] Taji T, Seki M, Satou M, Sakurai T, Kobayashi M, et al (2004) Comparative Genomics in Salt Tolerance between Arabidopsis and Arabidopsis-Related Halophyte Salt Cress Using Arabidopsis Microarray 1. Society 135: 1–13 doi:10.1104/pp.104.039909.1993. 10.1104/pp.104.039909 PMC519083 15247402

[22] Gong Q, Li P, Ma S, Indu Rupassara S, Bohnert HJ (2005) Salinity stress adaptation competence in the extremophile Thellungiella halophila in comparison with its relative Arabidopsis thaliana. The Plant journal: for cell and molecular biology 44: 826–839. 16297073 10.1111/j.1365-313X.2005.02587.x

[23] Wong CE, Li Y, Labbe A, Guevara D, Nuin P, et al (2006) Transcriptional Profiling Implicates Novel Interactions between Abiotic Stress and Hormonal Responses in Thellungiella, a Close Relative of Arabidopsis 1 [W]. Society 140: 1437–1450 doi:10.1104/pp.105.070508.1. 10.1104/pp.105.070508 PMC1435811 16500996

[24] Rabello AR, Guimarães CM, Rangel PHN, da Silva FR, Seixas D, et al (2008) Identification of drought-responsive genes in roots of upland rice (Oryza sativa L). BMC genomics 9: 485. 18922162 10.1186/1471-2164-9-485 PMC2605477

[25] Sun W, Xu X, Zhu H, Liu A, Liu L, et al (2010) Comparative transcriptomic profiling of a salt-tolerant wild tomato species and a salt-sensitive tomato cultivar. Plant & cell physiology 51: 997–1006. 20410049 10.1093/pcp/pcq056

[26] Cohen D, Bogeat-Triboulot M-B, Tisserant E, Balzergue S, Martin-Magniette M-L, et al (2010) Comparative transcriptomics of drought responses in Populus: a meta-analysis of genome-wide expression profiling in mature leaves and root apices across two genotypes. BMC genomics 11: 630. 21073700 10.1186/1471-2164-11-630 PMC3091765

[27] Mane S, Robinet C, Ulanov A, Schafleitner R, Tincopa L, et al (2008) Molecular and physiological adaptation to prolonged drought stress in the leaves of two Andean potato genotypes. Functional Plant Biology 35: 669–688. 10.1071/FP07293 32688822

[28] Rodrigues F, Delaia M, Zingaretti S (2009) Analysis of gene expression profiles under water stress in tolerant and sensitive sugarcane plants. Plant Science 176: 286–302.

[29] He X, Hou X, Shen Y, Huang Z (2011) TaSRG, a wheat transcription factor, significantly affects salt tolerance in transgenic rice and Arabidopsis. FEBS letters 585: 1231–1237. 21457711 10.1016/j.febslet.2011.03.055

[30] Chen W, Provart NJ, Glazebrook J, Katagiri F, Chang HS, et al (2002) Expression Profile Matrix of Arabidopsis Transcription Factor Genes Suggests Their Putative Functions in Response to Environmental Stresses. Society 14: 559–574 doi:10.1105/tpc.010410.560. 10.1105/tpc.010410 PMC150579 11910004

[31] Conde C, Silva P, Agasse A, Lemoine R, Delrot S, et al (2007) Utilization and transport of mannitol in Olea europaea and implications for salt stress tolerance. Plant & cell physiology 48: 42–53. 17118948 10.1093/pcp/pcl035

[32] Yu H, Gerstein M (2006) Genomic analysis of the hierarchical structure of regulatory networks. Proceedings of the National Academy of Sciences of the United States of America 103: 14724–14731. 17003135 10.1073/pnas.0508637103 PMC1595419

[33] Ihmels J, Levy R, Barkai N (2004) Principles of transcriptional control in the metabolic network of Saccharomyces cerevisiae. Nat Biotechnol 22: 86–92. 14647306 10.1038/nbt918

[34] Michoel T, De Smet R, Joshi A, Van de Peer Y, Marchal K (2009) Comparative analysis of module-based versus direct methods for reverse-engineering transcriptional regulatory networks. BMC systems biology 3: 49. 19422680 10.1186/1752-0509-3-49 PMC2684101

[35] Michoel T, De Smet R, Joshi A, Marchal K, Van de Peer Y (2009) Reverse-engineering transcriptional modules from gene expression data. Annals of the New York Academy of Sciences 1158: 36–43. 19348630 10.1111/j.1749-6632.2008.03943.x

[36] Vermeirssen V, Joshi A, Michoel T, Bonnet E, Casneuf T, et al (2009) Molecular BioSystems. Plant Biotechnology 5: 1817–1830 doi:10.1039/b908108a. 10.1039/B908108a 19763340

[37] Bonnet E, Tatari M, Joshi A, Michoel T, Marchal K, et al (2010) Module network inference from a cancer gene expression data set identifies microRNA regulated modules. PloS one 5: e10162. 20418949 10.1371/journal.pone.0010162 PMC2854686

[38] Conesa A, Götz S, García-Gómez JM, Terol J, Talón M, et al (2005) Blast2GO: a universal tool for annotation, visualization and analysis in functional genomics research. Bioinformatics (Oxford, England) 21: 3674–3676. 10.1093/bioinformatics/bti610 16081474

[39] Yang YH, Speed T (2002) Design issues for cDNA microarray experiments. Nature reviews Genetics 3: 579–588. 10.1038/nrg863 12154381

[40] Saeed AI, Sharov V, White J, Li J, Liang W, et al (2003) TM4: a free, open-source system for microarray data management and analysis. 34: 374–378. 10.2144/03342mt01 12613259

[41] Cramer GR, Urano K, Delrot S, Pezzotti M, Shinozaki K (2011) Effects of abiotic stress on plants: a systems biology perspective. BMC plant biology 11: 163. 22094046 10.1186/1471-2229-11-163 PMC3252258

[42] Michoel T, Maere S, Bonnet E, Joshi A, Saeys Y, et al (2007) Validating module network learning algorithms using simulated data. BMC bioinformatics 8 Suppl 2: S5. 10.1186/1471-2105-8-S2-S5 PMC1892074 17493254

[43] Wan X, Mo A, Liu S, Yang L, Li L (2011) Constitutive expression of a peanut ubiquitin-conjugating enzyme gene in Arabidopsis confers improved water-stress tolerance through regulation of stress-responsive gene expression. Journal of bioscience and bioengineering 111: 478–484. 21193345 10.1016/j.jbiosc.2010.11.021

[44] Al-Shahrour F, Minguez P, Tarraga J, Medina I, Alloza E, et al (2007) FatiGO +: a functional profiling tool for genomic data. Integration of functional annotation, regulatory motifs and interaction data with microarray experiments. Nucleic Acids Res 35: 91–96. 10.1093/nar/gkm260 PMC1933151 17478504

[45] Jakoby M, Weisshaar B, Dröge-Laser W, Vicente-Carbajosa J, Tiedemann J, et al (2002) bZIP transcription factors in Arabidopsis. Trends in plant science 7: 106–111. 11906833 10.1016/s1360-1385(01)02223-3

[46] Liao Y, Zou H-F, Wei W, Hao Y-J, Tian A-G, et al (2008) Soybean GmbZIP44, GmbZIP62 and GmbZIP78 genes function as negative regulator of ABA signaling and confer salt and freezing tolerance in transgenic Arabidopsis. Planta 228: 225–240. 18365246 10.1007/s00425-008-0731-3

[47] Bae MS, Cho EJ, Choi E-Y, Park OK (2003) Analysis of the Arabidopsis nuclear proteome and its response to cold stress. The Plant Journal 36: 652–663. 14617066 10.1046/j.1365-313x.2003.01907.x

[48] Stephenson TJ, McIntyre CL, Collet C, Xue G-P (2007) Genome-wide identification and expression analysis of the NF-Y family of transcription factors in Triticum aestivum. Plant molecular biology 65: 77–92. 17598077 10.1007/s11103-007-9200-9

[49] Liu J-X, Howell SH (2010) bZIP28 and NF-Y transcription factors are activated by ER stress and assemble into a transcriptional complex to regulate stress response genes in Arabidopsis. The Plant cell 22: 782–796. 20207753 10.1105/tpc.109.072173 PMC2861475

[50] Thön M, Al Abdallah Q, Hortschansky P, Scharf DH, Eisendle M, et al (2010) The CCAAT-binding complex coordinates the oxidative stress response in eukaryotes. Nucleic acids research 38: 1098–1113. 19965775 10.1093/nar/gkp1091 PMC2831313

[51] Hwang EW, Kim KA, Park SC, Jeong MJ, Byun MK, et al (2005) Expression profiles of hot pepper (Capsicum annuum) genes under cold stress conditions. J Biosci 30: 657–667. 16388140 10.1007/BF02703566

[52] Conti L, Price G, O'Donnell E, Schwessinger B, Dominy P, et al (2008) Small ubiquitin-like modifier proteases OVERLY TOLERANT TO SALT1 and -2 regulate salt stress responses in Arabidopsis. The Plant cell 20: 2894–2908. 18849491 10.1105/tpc.108.058669 PMC2590731

[53] Miura K, Jin JB, Hasegawa PM (2007) Sumoylation, a post-translational regulatory process in plants. Current Opinion in Plant Biology 10: 495–502. 17720613 10.1016/j.pbi.2007.07.002

[54] Munns R, Tester M (2008) Mechanisms of salinity tolerance. Annual review of plant biology 59: 651–681. 10.1146/annurev.arplant.59.032607.092911 18444910

[55] Bracci T, Busconi M, Fogher C, Sebastiani L (2011) Molecular studies in olive (Olea europaea L.): overview on DNA markers applications and recent advances in genome analysis. Plant cell reports 30: 449–462. 21212959 10.1007/s00299-010-0991-9

[56] Golldack D, Lüking I, Yang O (2011) Plant tolerance to drought and salinity: stress regulating transcription factors and their functional significance in the cellular transcriptional network. Plant cell reports 1383–1391. 21476089 10.1007/s00299-011-1068-0

[57] Zhang H, Huang Z, Xie B, Chen Q, Tian X, et al (2004) The ethylene-, jasmonate-, abscisic acid- and NaCl-responsive tomato transcription factor JERF1 modulates expression of GCC box-containing genes and salt tolerance in tobacco. Planta 220: 262–270. 15300440 10.1007/s00425-004-1347-x

[58] Yang O, Popova OV, Süthoff U, Lüking I, Dietz K-J, et al (2009) The Arabidopsis basic leucine zipper transcription factor AtbZIP24 regulates complex transcriptional networks involved in abiotic stress resistance. Gene 436: 45–55. 19248824 10.1016/j.gene.2009.02.010

[59] Yoshida T, Fujita Y, Sayama H, Kidokoro S, Maruyama K, et al (2010) AREB1, AREB2, and ABF3 are master transcription factors that cooperatively regulate ABRE-dependent ABA signaling involved in drought stress tolerance and require ABA for full activation. Plant Journal 61: 672–685. 19947981 10.1111/j.1365-313X.2009.04092.x

[60] Nelson DE, Repetti PP, Adams TR, Creelman RA, Wu J, et al (2007) Plant nuclear factor Y (NF-Y) B subunits confer drought tolerance and lead to improved corn yields on water-limited acres. Proceedings of the National Academy of Sciences of the United States of America 104: 16450–16455. 17923671 10.1073/pnas.0707193104 PMC2034233

[61] Hoagland DR, Arnon DI (1950) The water-culture method for growing plants without soil. California Agricultural Experiment Station Circular 347: 1–32.

[62] Sioson AA, Mane SP, Li P, Sha W, Heath LS, et al (2006) The statistics of identifying differentially expressed genes in Expresso and TM4: a comparison. BMC Bioinformatics 7: 215. 16626497 10.1186/1471-2105-7-215 PMC1513403

[63] Quackenbush J (2002) Microarray data normalization and transformation. Nat Genet 32: 496–501. 12454644 10.1038/ng1032

[64] Tusher VG, Tibshirani R, Chu G (2001) Significance analysis of microarrays applied to the ionizing radiation response. Proc Natl Acad Sci U S A 98: 5116–5121. 11309499 10.1073/pnas.091062498 PMC33173

[65] Bonnet E, Michoel T, Van de Peer Y (2010) Prediction of a gene regulatory network linked to prostate cancer from gene expression, microRNA and clinical data. Bioinformatics (Oxford, England) 26: i638–44. 10.1093/bioinformatics/btq395 PMC2935430 20823333

[66] Labarga A, Valentin F, Anderson M, Lopez R (2007) Web services at the European bioinformatics institute. Nucleic Acids Res 35. 10.1093/nar/gkm291 PMC1933145 17576686

[67] Shannon P, Markiel A, Ozier O, Baliga NS, Wang JT, et al (2003) Cytoscape: a software environment for integrated models of biomolecular interaction networks. Genome Res 13: 2498–2504. 14597658 10.1101/gr.1239303 PMC403769

[68] Assenov Y, Ramirez F, Schelhorn SE, Lengauer T, Albrecht M (2008) Computing topological parameters of biological networks. Bioinformatics 24: 282–284. 18006545 10.1093/bioinformatics/btm554

