# Supplementary figures and images for: Comparative Transcriptome Analysis of Two Olive Cultivars in Response to NaCl-Stress
Source: PLoS One. 2012 Aug 30;7(8):e42931. doi: 10.1371/journal.pone.0042931 (PMC3431368; doi:10.1371/journal.pone.0042931)

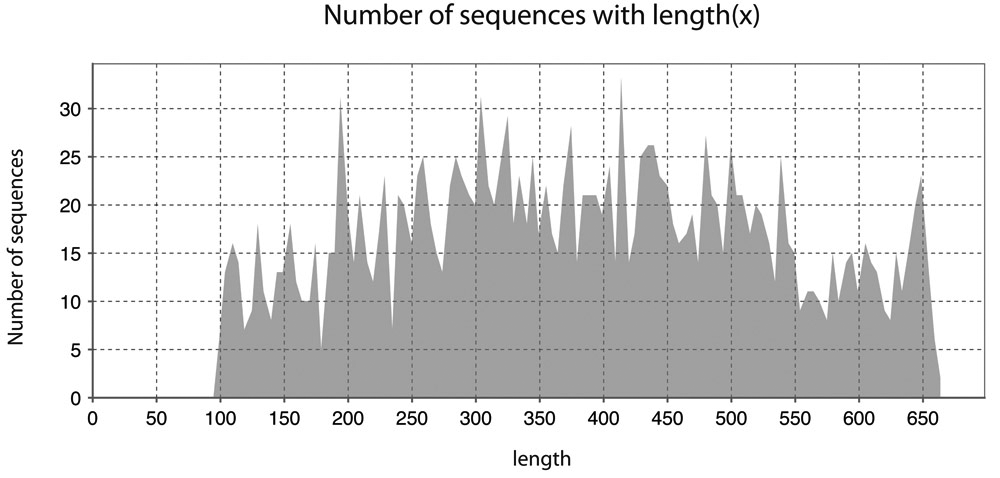

Supplement: Figure S1 — Distribution of sequence length for the 1956 ESTs. (TIF) [file pone.0042931.s001.tif]

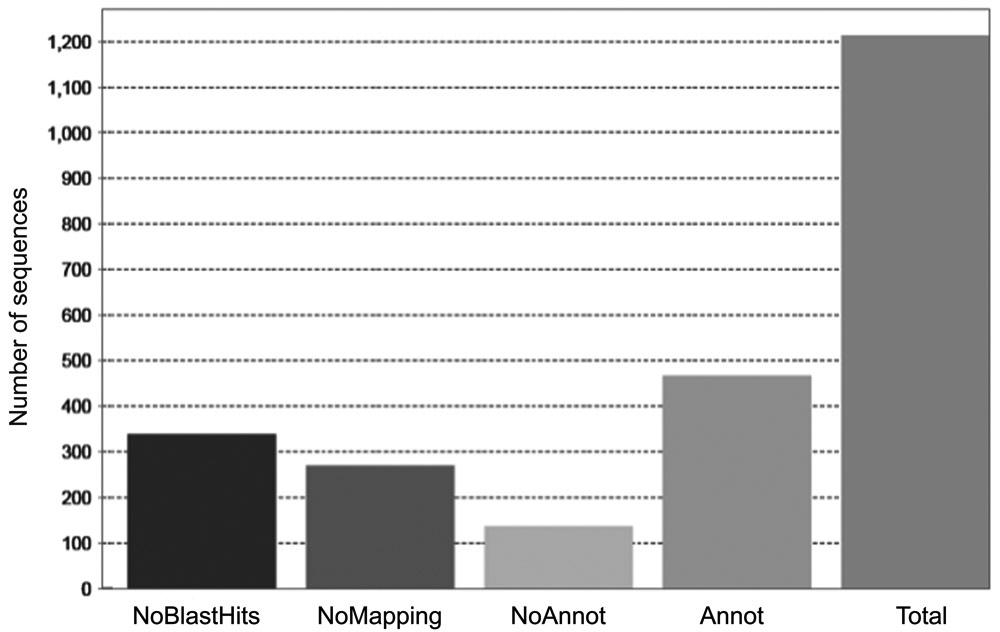

Supplement: Figure S2 — Distributions of the blastx analysis of the 1211 non-redundant ESTs. NoBlastHits: sequences returning no blast hits, NoMapping: Mapping step returned no results for the sequence, NoAnnot: Annotation step returned no results for the sequence, Annot: Sequences with a GO assignment, Total: total number of sequences. (TIF) [file pone.0042931.s002.tif]

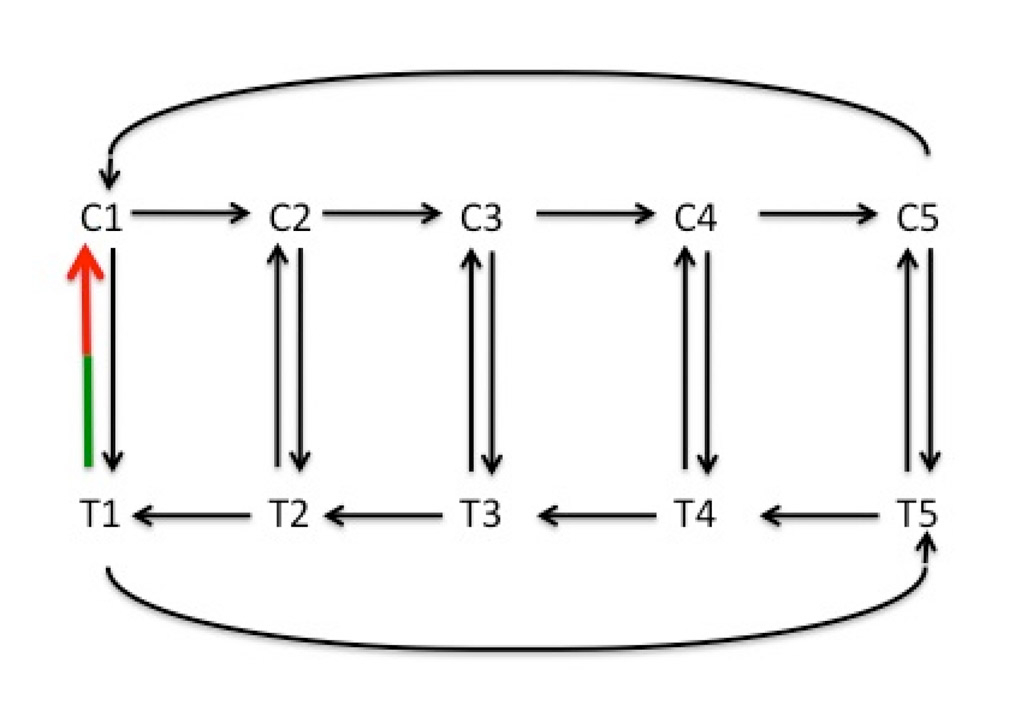

Supplement: Figure S3 — Loop design of the time course experiment. C: Control; T: NaCl-treatement; 1: 15 days stress; 2: 45 days stress; 3: 90 days stress; 4: 15 days post-stress; 5: 45 days post-stress. Each arrow represents a two-dye hybridization pair among the experimental treatments. Every time point sample was hybridized four times, each time with RNA extracted from a different plant. The head and tail of each arrow represent the Alexa 647 and Alexa 555 dye respectively. (TIF) [file pone.0042931.s003.tif]

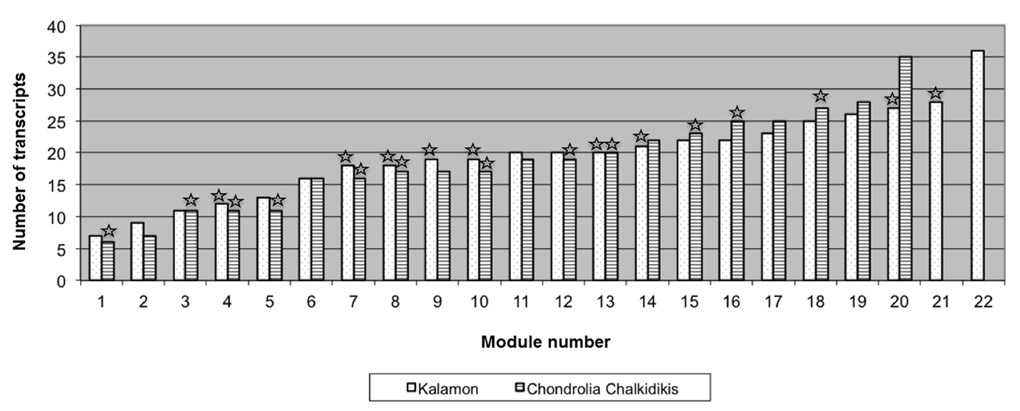

Supplement: Figure S4 — Transcript distribution in regulatory modules for cv. Kalamon and cv. Chondrolia Chalkidikis. The Kalamon network consists of 22 modules (white bars) comprising 432 transcripts. Nine of the modules marked with a star comprise 186 transcripts and are regulated by at least one transcription factor. The Chondrolia Chalkidikis network consists of 20 modules (stripped bars) comprising 372 transcripts. Twelve of the modules marked with a star comprise 237 transcripts and are regulated by at least one transcription factor. (TIF) [file pone.0042931.s004.tif]
