# Supplementary material for: Comparative Transcriptome Analysis of Two Olive Cultivars in Response to NaCl-Stress
Source: PLoS One. 2012 Aug 30;7(8):e42931. doi: 10.1371/journal.pone.0042931 (PMC3431368; doi:10.1371/journal.pone.0042931)
Supplement: Table S1 — Redundancy level of the olive seedling cDNA library. The first column shows the number of ESTs that comprise a contig while the second column shows the total number of contigs with the corresponding number of ESTs. (DOCX) [file pone.0042931.s005.docx]

**Supplementary Table 1. Redundancy level of the olive seedling cDNA library.**

| **Total number of ESTs/contig** | **Total number of contigs** |
| --- | --- |
| 2-4 | 185 |
| 5-10 | 16 |
| 11-20 | 6 |
| 21-50 | 5 |
| 51-100 | 3 |

The first column shows the number of ESTs that comprise a contig while the second column shows the total number of contigs with the corresponding number of ESTs.
