# Supplementary material for: Comparative Transcriptome Analysis of Two Olive Cultivars in Response to NaCl-Stress
Source: PLoS One. 2012 Aug 30;7(8):e42931. doi: 10.1371/journal.pone.0042931 (PMC3431368; doi:10.1371/journal.pone.0042931)
Supplement: Table S2 — GO categories for clusters of the differentially expressed transcripts in cv. Kalamon. Transcript name: The name of the transcript in a cluster. Hit description: The description of the GO term assigned to the transcript. GO-ID: The GO-ID term assigned to the transcript. Term: The GO term assigned to the transcript. (DOCX) [file pone.0042931.s006.docx]

### Supplementary Table 2. GO categories for clusters of the differentially expressed transcripts in cv. Kalamon.

| **Cluster A** | | | |
| --- | --- | --- | --- |
| **Transcript name** | **Hit description** | **GO-ID** | **Term** |
| se001_B11 | 1-aminocyclopropane-1-carboxylate oxidase | GO:0055114 | oxidation reduction |
|  | 1-aminocyclopropane-1-carboxylate oxidase | GO:0009815 | 1-aminocyclopropane-1-carboxylate oxidase activity |
| se007_E3 | chorismate mutase | GO:0004106 | chorismate mutase activity |
|  | chorismate mutase | GO:0009073 | aromatic amino acid family biosynthetic process |
| se023_D8 | multiple stress-responsive zinc-finger protein | GO:0008270 | zinc ion binding |
| se015_G7 | phospholipase d | GO:0016049 | cell growth |
|  | phospholipase d | GO:0045848 | positive regulation of nitrogen utilization |
|  | phospholipase d | GO:0048364 | root development |
|  | phospholipase d | GO:0009395 | phospholipid catabolic process |
|  | phospholipase d | GO:0006643 | membrane lipid metabolic process |
|  | phospholipase d | GO:0051301 | cell division |
|  | phospholipase d | GO:0051365 | cellular response to potassium ion starvation |
|  | phospholipase d | GO:0004630 | phospholipase D activity |
|  | phospholipase d | GO:0006970 | response to osmotic stress |
|  | phospholipase d | GO:0009791 | post-embryonic development |
|  | phospholipase d | GO:0006995 | cellular response to nitrogen starvation |
|  | phospholipase d | GO:0016036 | cellular response to phosphate starvation |
|  | phospholipase d | GO:0005886 | plasma membrane |
| se016_D11 | sucrose phosphate synthase | GO:0008152 | metabolic process |
|  | sucrose phosphate synthase | GO:0016757 | transferase activity, transferring glycosyl groups |
| se022_G8 | NA | GO:0008121 | ubiquinol-cytochrome-c reductase activity |
| se023_D4 | dsrna-binding protein | GO:0015030 | Cajal body |
|  | dsrna-binding protein | GO:0010445 | nuclear dicing body |
|  | dsrna-binding protein | GO:0009735 | response to cytokinin stimulus |
|  | dsrna-binding protein | GO:0035196 | production of miRNAs involved in gene silencing by miRNA |
|  | dsrna-binding protein | GO:0035279 | mRNA cleavage involved in gene silencing by miRNA |
|  | dsrna-binding protein | GO:0035198 | miRNA binding |
|  | dsrna-binding protein | GO:0042802 | identical protein binding |
|  | dsrna-binding protein | GO:0010267 | production of ta-siRNAs involved in RNA interference |
|  | dsrna-binding protein | GO:0003725 | double-stranded RNA binding |
|  | dsrna-binding protein | GO:0009733 | response to auxin stimulus |
|  | dsrna-binding protein | GO:0009737 | response to abscisic acid stimulus |
| se013_B12 | one helix protein 2 | GO:0009642 | response to light intensity |
|  | one helix protein 2 | GO:0009535 | chloroplast thylakoid membrane |
| **Cluster B** | | | |
| **Transcript Name** | **Hit-Description** | **GO-ID** | **Term** |
| se011_A8 | ubiquitin-associated ts-n domain-containing protein | GO:0005622 | intracellular |
|  | ubiquitin-associated ts-n domain-containing protein | GO:0008270 | zinc ion binding |
| se015_B4 | glycoside hydrolase family 28 protein | GO:0044464 | cell part |
|  | glycoside hydrolase family 28 protein | GO:0004650 | polygalacturonase activity |
|  | glycoside hydrolase family 28 protein | GO:0005975 | carbohydrate metabolic process |
| **Cluster C** | | | |
| **Transcript Name** | **Hit-Description** | **GO-ID** | **Term** |
| se023_E3 | mitochondrial import inner membrane translocase subunit tim9 | GO:0055085 | transmembrane transport |
|  | mitochondrial import inner membrane translocase subunit tim9 | GO:0015450 | P-P-bond-hydrolysis-driven protein transmembrane transporter activity |
|  | mitochondrial import inner membrane translocase subunit tim9 | GO:0009793 | embryonic development ending in seed dormancy |
|  | mitochondrial import inner membrane translocase subunit tim9 | GO:0005743 | mitochondrial inner membrane |
|  | mitochondrial import inner membrane translocase subunit tim9 | GO:0046872 | metal ion binding |
|  | mitochondrial import inner membrane translocase subunit tim9 | GO:0045039 | protein import into mitochondrial inner membrane |
|  | mitochondrial import inner membrane translocase subunit tim9 | GO:0042719 | mitochondrial intermembrane space protein transporter complex |
| se013_D9 | mal d | GO:0006952 | defense response |
|  | mal d | GO:0009607 | response to biotic stimulus |
| se011_B7 | pollen ole e 1 allergen and extensin family protein | GO:0044464 | cell part |
| se007_E10 | hect ubiquitin-protein ligase 3 | GO:0042023 | DNA endoreduplication |
|  | hect ubiquitin-protein ligase 3 | GO:0005488 | binding |
|  | hect ubiquitin-protein ligase 3 | GO:0006464 | protein modification process |
|  | hect ubiquitin-protein ligase 3 | GO:0005622 | intracellular |
|  | hect ubiquitin-protein ligase 3 | GO:0010091 | trichome branching |
|  | hect ubiquitin-protein ligase 3 | GO:0004842 | ubiquitin-protein ligase activity |
| se011_A4 | nuclear inhibitor of protein phosphatase- | GO:0048638 | regulation of developmental growth |
|  | nuclear inhibitor of protein phosphatase- | GO:0008283 | cell proliferation |
|  | nuclear inhibitor of protein phosphatase- | GO:0003723 | RNA binding |
|  | nuclear inhibitor of protein phosphatase- | GO:0005515 | protein binding |
|  | nuclear inhibitor of protein phosphatase- | GO:0035196 | production of miRNAs involved in gene silencing by miRNA |
|  | nuclear inhibitor of protein phosphatase- | GO:0005634 | nucleus |
|  | nuclear inhibitor of protein phosphatase- | GO:0005829 | cytosol |
| se015_E4 | 26s proteasome regulatory subunit | GO:0005852 | eukaryotic translation initiation factor 3 complex |
|  | 26s proteasome regulatory subunit | GO:0006413 | translational initiation |
|  | 26s proteasome regulatory subunit | GO:0003743 | translation initiation factor activity |
|  | 26s proteasome regulatory subunit | GO:0016020 | membrane |
|  | 26s proteasome regulatory subunit | GO:0005634 | nucleus |
| se016_A11 | rcd1 (radical-induced cell death1) protein binding | GO:0012501 | programmed cell death |
|  | rcd1 (radical-induced cell death1) protein binding | GO:0010102 | lateral root morphogenesis |
|  | rcd1 (radical-induced cell death1) protein binding | GO:0010193 | response to ozone |
|  | rcd1 (radical-induced cell death1) protein binding | GO:0009414 | response to water deprivation |
|  | rcd1 (radical-induced cell death1) protein binding | GO:0009867 | jasmonic acid mediated signaling pathway |
|  | rcd1 (radical-induced cell death1) protein binding | GO:0009651 | response to salt stress |
|  | rcd1 (radical-induced cell death1) protein binding | GO:0009790 | embryonic development |
|  | rcd1 (radical-induced cell death1) protein binding | GO:0005515 | protein binding |
|  | rcd1 (radical-induced cell death1) protein binding | GO:0000303 | response to superoxide |
|  | rcd1 (radical-induced cell death1) protein binding | GO:0005737 | cytoplasm |
|  | rcd1 (radical-induced cell death1) protein binding | GO:0042542 | response to hydrogen peroxide |
|  | rcd1 (radical-induced cell death1) protein binding | GO:0006809 | nitric oxide biosynthetic process |
|  | rcd1 (radical-induced cell death1) protein binding | GO:0009816 | defense response to bacterium, incompatible interaction |
|  | rcd1 (radical-induced cell death1) protein binding | GO:0005634 | nucleus |
|  | rcd1 (radical-induced cell death1) protein binding | GO:0009873 | ethylene mediated signaling pathway |
| se015_D5 | protein | GO:0009941 | chloroplast envelope |
|  | protein | GO:0019538 | protein metabolic process |
|  | protein | GO:0005515 | protein binding |
|  | protein | GO:0005524 | ATP binding |
|  | protein | GO:0009570 | chloroplast stroma |
|  | protein | GO:0009579 | thylakoid |
| se014_H11 | 3-deoxy-d-arabino-heptulosonate 7-phosphate synthase | GO:0033587 | shikimate biosynthetic process |
|  | 3-deoxy-d-arabino-heptulosonate 7-phosphate synthase | GO:0009423 | chorismate biosynthetic process |
|  | 3-deoxy-d-arabino-heptulosonate 7-phosphate synthase | GO:0009073 | aromatic amino acid family biosynthetic process |
|  | 3-deoxy-d-arabino-heptulosonate 7-phosphate synthase | GO:0016020 | membrane |
|  | 3-deoxy-d-arabino-heptulosonate 7-phosphate synthase | GO:0009536 | plastid |
|  | 3-deoxy-d-arabino-heptulosonate 7-phosphate synthase | GO:0003849 | 3-deoxy-7-phosphoheptulonate synthase activity |
| se022_A6 | ghmp kinase family protein | GO:0044237 | cellular metabolic process |
|  | ghmp kinase family protein | GO:0016772 | transferase activity, transferring phosphorus-containing groups |
|  | ghmp kinase family protein | GO:0000166 | nucleotide binding |
| se015_A5 | nadh-ubiquinone oxidoreductase b18 | GO:0006120 | mitochondrial electron transport, NADH to ubiquinone |
|  | nadh-ubiquinone oxidoreductase b18 | GO:0045271 | respiratory chain complex I |
|  | nadh-ubiquinone oxidoreductase b18 | GO:0008137 | NADH dehydrogenase (ubiquinone) activity |
|  | nadh-ubiquinone oxidoreductase b18 | GO:0009853 | photorespiration |
|  | nadh-ubiquinone oxidoreductase b18 | GO:0009536 | plastid |
|  | nadh-ubiquinone oxidoreductase b18 | GO:0031966 | mitochondrial membrane |
| se011_D4 | chaperone protein | GO:0006950 | response to stress |
|  | chaperone protein | GO:0005634 | nucleus |
|  | chaperone protein | GO:0031072 | heat shock protein binding |
| se007_G9 | ccr4-associated factor | GO:0004535 | poly(A)-specific ribonuclease activity |
|  | ccr4-associated factor | GO:0003676 | nucleic acid binding |
|  | ccr4-associated factor | GO:0005634 | nucleus |
|  | ccr4-associated factor | GO:0009451 | RNA modification |
| **Cluster D** | | | |
| **Transcript Name** | **Hit-Description** | **GO-ID** | **Term** |
| se015_B12 | gras family transcription factor | GO:0009739 | response to gibberellin stimulus |
|  | gras family transcription factor | GO:0045449 | regulation of transcription |
|  | gras family transcription factor | GO:0003700 | transcription factor activity |
|  | gras family transcription factor | GO:0005634 | nucleus |
| se015_A1 | small glutamine-rich tetratricopeptide repeat-containing protein | GO:0009651 | response to salt stress |
|  | small glutamine-rich tetratricopeptide repeat-containing protein | GO:0009789 | positive regulation of abscisic acid mediated signaling pathway |
| se015_D11 | multidrug pheromone mdr abc transporter family | GO:0009987 | cellular process |
|  | multidrug pheromone mdr abc transporter family | GO:0010540 | basipetal auxin transport |
|  | multidrug pheromone mdr abc transporter family | GO:0009958 | positive gravitropism |
|  | multidrug pheromone mdr abc transporter family | GO:0042626 | ATPase activity, coupled to transmembrane movement of substances |
|  | multidrug pheromone mdr abc transporter family | GO:0009640 | photomorphogenesis |
|  | multidrug pheromone mdr abc transporter family | GO:0009637 | response to blue light |
|  | multidrug pheromone mdr abc transporter family | GO:0010541 | acropetal auxin transport |
|  | multidrug pheromone mdr abc transporter family | GO:0009733 | response to auxin stimulus |
|  | multidrug pheromone mdr abc transporter family | GO:0008361 | regulation of cell size |
|  | multidrug pheromone mdr abc transporter family | GO:0010329 | auxin efflux transmembrane transporter activity |
|  | multidrug pheromone mdr abc transporter family | GO:0043481 | anthocyanin accumulation in tissues in response to UV light |
|  | multidrug pheromone mdr abc transporter family | GO:0005515 | protein binding |
|  | multidrug pheromone mdr abc transporter family | GO:0048443 | stamen development |
|  | multidrug pheromone mdr abc transporter family | GO:0048527 | lateral root development |
|  | multidrug pheromone mdr abc transporter family | GO:0010218 | response to far red light |
|  | multidrug pheromone mdr abc transporter family | GO:0005886 | plasma membrane |
|  | multidrug pheromone mdr abc transporter family | GO:0005524 | ATP binding |
| se021_H12 | amp-dependent synthetase and ligase | GO:0009507 | chloroplast |
|  | amp-dependent synthetase and ligase | GO:0042372 | phylloquinone biosynthetic process |
|  | amp-dependent synthetase and ligase | GO:0008756 | o-succinylbenzoate-CoA ligase activity |
| se023_B3 | act domain-containing protein | GO:0009941 | chloroplast envelope |
|  | act domain-containing protein | GO:0009409 | response to cold |
|  | act domain-containing protein | GO:0016597 | amino acid binding |
|  | act domain-containing protein | GO:0010319 | stromule |
|  | act domain-containing protein | GO:0009570 | chloroplast stroma |
|  | act domain-containing protein | GO:0008152 | metabolic process |
|  | act domain-containing protein | GO:0009535 | chloroplast thylakoid membrane |
| se013_G10 | inosine-5 -monophosphate dehydrogenase | GO:0003938 | IMP dehydrogenase activity |
|  | inosine-5 -monophosphate dehydrogenase | GO:0006177 | GMP biosynthetic process |
|  | inosine-5 -monophosphate dehydrogenase | GO:0046872 | metal ion binding |
|  | inosine-5 -monophosphate dehydrogenase | GO:0055114 | oxidation reduction |
| se022_F6 | hexokinase 2 | GO:0012501 | programmed cell death |
|  | hexokinase 2 | GO:0009527 | plastid outer membrane |
|  | hexokinase 2 | GO:0005739 | mitochondrion |
|  | hexokinase 2 | GO:0005975 | carbohydrate metabolic process |
|  | hexokinase 2 | GO:0005524 | ATP binding |
|  | hexokinase 2 | GO:0009747 | hexokinase-dependent signaling |
|  | hexokinase 2 | GO:0009507 | chloroplast |
|  | hexokinase 2 | GO:0004340 | glucokinase activity |
|  | hexokinase 2 | GO:0044237 | cellular metabolic process |
|  | hexokinase 2 | GO:0008865 | fructokinase activity |

Transcript name: The name of the transcript in a cluster. Hit description: The description of the GO term assigned to the transcript. GO-ID: The GO-ID term assigned to the transcript. Term: The GO term assigned to the transcript. NA: Not Available
