# Supplementary material for: Comparative Transcriptome Analysis of Two Olive Cultivars in Response to NaCl-Stress
Source: PLoS One. 2012 Aug 30;7(8):e42931. doi: 10.1371/journal.pone.0042931 (PMC3431368; doi:10.1371/journal.pone.0042931)
Supplement: Table S3 — GO categories for modules that comprise cv. Kalamon transcriptional regulatory network. The first column shows the module number, the second column shows the number of transcripts that comprise the module. Columns three and four show the GO ID and GO Term respectively while the last column shows the p-value of the GO term assignment as calculated in the FatiGO tool. (DOCX) [file pone.0042931.s007.docx]

**Supplementary Table 3.** **GO categories for modules that comprise cv. Kalamon transcriptional regulatory network.**

| **Module ID** | **Number of transcripts** | **GO-ID** | **Term** | **P-Value** |
| --- | --- | --- | --- | --- |
| 1 | 19 | GO:0040007 | growth | 1.71E-03 |
|  |  | GO:0004535 | poly(A)-specific ribonuclease activity | 9.63E-03 |
|  |  | GO:0016796 | exonuclease activity, active with either ribo- or deoxyribonucleic acids and producing 5'-phosphomonoesters | 9.63E-03 |
|  |  | GO:0004532 | exoribonuclease activity | 9.63E-03 |
|  |  | GO:0004527 | exonuclease activity | 9.63E-03 |
|  |  | GO:0016896 | exoribonuclease activity, producing 5'-phosphomonoesters | 9.63E-03 |
|  |  | GO:0008408 | 3'-5' exonuclease activity | 9.63E-03 |
|  |  | GO:0000175 | 3'-5'-exoribonuclease activity | 9.63E-03 |
|  |  | GO:0004758 | serine C-palmitoyltransferase activity | 9.63E-03 |
|  |  | GO:0016409 | palmitoyltransferase activity | 9.63E-03 |
|  |  | GO:0016454 | C-palmitoyltransferase activity | 9.63E-03 |
|  |  | GO:0030148 | sphingolipid biosynthetic process | 9.63E-03 |
|  |  | GO:0046467 | membrane lipid biosynthetic process | 9.63E-03 |
|  |  | GO:0009051 | pentose-phosphate shunt, oxidative branch | 9.63E-03 |
|  |  | GO:0004345 | glucose-6-phosphate dehydrogenase activity | 9.63E-03 |
|  |  | GO:0015297 | antiporter activity | 9.63E-03 |
|  |  | GO:0008283 | cell proliferation | 9.63E-03 |
| 2 | 13 | GO:0046872 | metal ion binding | 2.24E-04 |
|  |  | GO:0043169 | cation binding | 2.85E-04 |
|  |  | GO:0043167 | ion binding | 2.85E-04 |
|  |  | GO:0010038 | response to metal ion | 9.17E-03 |
| 3 | 16 | GO:0009507 | chloroplast | 2.95E-03 |
| 4 | 20 | No GOs are overrepresented | | |
| 5 | 22 | GO:0006259 | DNA metabolic process | 2.94E-03 |
|  |  | GO:0016779 | nucleotidyltransferase activity | 3.93E-03 |
|  |  | GO:0004484 | mRNA guanylyltransferase activity | 6.74E-03 |
|  |  | GO:0008192 | RNA guanylyltransferase activity | 6.74E-03 |
|  |  | GO:0006370 | mRNA capping | 6.74E-03 |
|  |  | GO:0009452 | RNA capping | 6.74E-03 |
|  |  | GO:0006278 | RNA-dependent DNA replication | 6.74E-03 |
|  |  | GO:0003964 | RNA-directed DNA polymerase activity | 6.74E-03 |
| 6 | 18 | No GOs are overrepresented | | |
| 7 | 25 | GO:0009723 | response to ethylene stimulus | 2.46E-03 |
|  |  | GO:0009889 | regulation of biosynthetic process | 9.14E-03 |
|  |  | GO:0031326 | regulation of cellular biosynthetic process | 9.14E-03 |
|  |  | GO:0010556 | regulation of macromolecule biosynthetic process | 9.14E-03 |
| 8 | 23 | GO:0016020 | membrane | 2.46E-03 |
|  |  | GO:0031090 | organelle membrane | 5.02E-03 |
|  |  | GO:0044425 | membrane part | 6.55E-03 |
|  |  | GO:0009579 | thylakoid | 7.35E-03 |
|  |  | GO:0044446 | intracellular organelle part | 9.52E-03 |
|  |  | GO:0044422 | organelle part | 9.52E-03 |
| 9 | 18 | GO:0050896 | response to stimulus | 1.75E-03 |
| 10 | 26 | GO:0043094 | cellular metabolic compound salvage | 5.06E-03 |
|  |  | GO:0009266 | response to temperature stimulus | 7.52E-03 |
|  |  | GO:0009628 | response to abiotic stimulus | 9.22E-03 |
| 11 | 28 | No GOs are overrepresented | | |
| 12 | 27 | No GOs are overrepresented | | |
| 13 | 19 | GO:0004028 | 3-chloroallyl aldehyde dehydrogenase activity | 9.63E-03 |
|  |  | GO:0045040 | protein import into mitochondrial outer membrane | 9.63E-03 |
|  |  | GO:0007008 | outer mitochondrial membrane organization | 9.63E-03 |
|  |  | GO:0005742 | mitochondrial outer membrane translocase complex | 9.63E-03 |
|  |  | GO:0005744 | mitochondrial inner membrane presequence translocase complex | 9.63E-03 |
|  |  | GO:0010252 | auxin homeostasis | 9.63E-03 |
|  |  | GO:0009860 | pollen tube growth | 9.63E-03 |
|  |  | GO:0048610 | reproductive cellular process | 9.63E-03 |
|  |  | GO:0001558 | regulation of cell growth | 9.63E-03 |
|  |  | GO:0010053 | root epidermal cell differentiation | 9.63E-03 |
|  |  | GO:0010054 | trichoblast differentiation | 9.63E-03 |
|  |  | GO:0021700 | developmental maturation | 9.63E-03 |
|  |  | GO:0048469 | cell maturation | 9.63E-03 |
|  |  | GO:0048764 | trichoblast maturation | 9.63E-03 |
|  |  | GO:0048765 | root hair cell differentiation | 9.63E-03 |
|  |  | GO:0048588 | developmental cell growth | 9.63E-03 |
|  |  | GO:0048767 | root hair elongation | 9.63E-03 |
|  |  | GO:0048768 | root hair cell tip growth | 9.63E-03 |
|  |  | GO:0060560 | developmental growth involved in morphogenesis | 9.63E-03 |
|  |  | GO:0009826 | unidimensional cell growth | 9.63E-03 |
|  |  | GO:0009932 | cell tip growth | 9.63E-03 |
|  |  | GO:0008514 | organic anion transmembrane transporter activity | 9.63E-03 |
| 14 | 36 | GO:0048856 | anatomical structure development | 1.05E-03 |
|  |  | GO:0090066 | regulation of anatomical structure size | 3.41E-03 |
|  |  | GO:0032535 | regulation of cellular component size | 3.41E-03 |
|  |  | GO:0008361 | regulation of cell size | 3.41E-03 |
|  |  | GO:0007275 | multicellular organismal development | 4.65E-03 |
|  |  | GO:0016887 | ATPase activity | 4.87E-03 |
|  |  | GO:0042623 | ATPase activity, coupled | 4.87E-03 |
|  |  | GO:0032501 | multicellular organismal process | 5.74E-03 |
|  |  | GO:0016757 | transferase activity, transferring glycosyl groups | 6.53E-03 |
|  |  | GO:0048513 | organ development | 6.53E-03 |
|  |  | GO:0008026 | ATP-dependent helicase activity | 6.99E-03 |
|  |  | GO:0004386 | helicase activity | 6.99E-03 |
|  |  | GO:0070035 | purine NTP-dependent helicase activity | 6.99E-03 |
|  |  | GO:0048731 | system development | 7.47E-03 |
|  |  | GO:0009791 | post-embryonic development | 7.63E-03 |
|  |  | GO:0032502 | developmental process | 8.45E-03 |
|  |  | GO:0022622 | root system development | 9.22E-03 |
|  |  | GO:0048364 | root development | 9.22E-03 |
| 15 | 20 | GO:0030529 | ribonucleoprotein complex | 9.89E-03 |
| 16 | 20 | GO:0005680 | anaphase-promoting complex | 9.63E-03 |
|  |  | GO:0000152 | nuclear ubiquitin ligase complex | 9.63E-03 |
|  |  | GO:0010229 | inflorescence development | 9.63E-03 |
|  |  | GO:0046470 | phosphatidylcholine metabolic process | 9.63E-03 |
|  |  | GO:0046486 | glycerolipid metabolic process | 9.63E-03 |
|  |  | GO:0042439 | ethanolamine and derivative metabolic process | 9.63E-03 |
|  |  | GO:0006650 | glycerophospholipid metabolic process | 9.63E-03 |
|  |  | GO:0070290 | NAPE-specific phospholipase D activity | 9.63E-03 |
|  |  | GO:0015175 | neutral amino acid transmembrane transporter activity | 9.63E-03 |
|  |  | GO:0015172 | acidic amino acid transmembrane transporter activity | 9.63E-03 |
|  |  | GO:0005275 | amine transmembrane transporter activity | 9.63E-03 |
|  |  | GO:0015171 | amino acid transmembrane transporter activity | 9.63E-03 |
| 17 | 22 | No GOs are overrepresented | | |
| 18 | 11 | GO:0006725 | cellular aromatic compound metabolic process | 1.07E-03 |
|  |  | GO:0010337 | regulation of salicylic acid metabolic process | 6.74E-03 |
|  |  | GO:0010565 | regulation of cellular ketone metabolic process | 6.74E-03 |
|  |  | GO:0009696 | salicylic acid metabolic process | 6.74E-03 |
|  |  | GO:0019789 | SUMO ligase activity | 6.74E-03 |
|  |  | GO:0050737 | O-hydroxycinnamoyltransferase activity | 6.74E-03 |
|  |  | GO:0050734 | hydroxycinnamoyltransferase activity | 6.74E-03 |
| 19 | 21 | No GOs are overrepresented | | |
| 20 | 7 | GO:0006012 | galactose metabolic process | 3.85E-03 |
|  |  | GO:0003978 | UDP-glucose 4-epimerase activity | 3.85E-03 |
|  |  | GO:0016857 | racemase and epimerase activity, acting on carbohydrates and derivatives | 3.85E-03 |
|  |  | GO:0006406 | mRNA export from nucleus | 3.85E-03 |
|  |  | GO:0006403 | RNA localization | 3.85E-03 |
|  |  | GO:0051236 | establishment of RNA localization | 3.85E-03 |
|  |  | GO:0050657 | nucleic acid transport | 3.85E-03 |
|  |  | GO:0051168 | nuclear export | 3.85E-03 |
|  |  | GO:0050658 | RNA transport | 3.85E-03 |
|  |  | GO:0051028 | mRNA transport | 3.85E-03 |
|  |  | GO:0006405 | RNA export from nucleus | 3.85E-03 |
|  |  | GO:0031965 | nuclear membrane | 3.85E-03 |
|  |  | GO:0000300 | peripheral to membrane of membrane fraction | 3.85E-03 |
|  |  | GO:0006888 | ER to Golgi vesicle-mediated transport | 7.70E-03 |
|  |  | GO:0048193 | Golgi vesicle transport | 7.70E-03 |
|  |  | GO:0015931 | nucleobase, nucleoside, nucleotide and nucleic acid transport | 7.70E-03 |
| 21 | 12 | No GOs are overrepresented | | |
| 22 | 9 | GO:0050832 | defense response to fungus | 8.65E-03 |

The first column shows the module number, the second column shows the number of transcripts that comprise the module. Columns three and four show the GO ID and GO Term respectively while the last column shows the *p-*value of the GO term assignment as calculated in FatiGO tool.
