# Supplementary material for: Comparative Transcriptome Analysis of Two Olive Cultivars in Response to NaCl-Stress
Source: PLoS One. 2012 Aug 30;7(8):e42931. doi: 10.1371/journal.pone.0042931 (PMC3431368; doi:10.1371/journal.pone.0042931)
Supplement: Table S4 — GO categories for modules that comprise cv. Chondrolia Chalkidikis transcriptional regulatory network. The first column shows the module number, the second column shows the number of transcripts that comprise the module. Columns three and four show the GO ID and GO Term respectively while the last column shows the p-value of the GO term assignment as calculated in the FatiGO tool. (DOCX) [file pone.0042931.s008.docx]

**Supplementary Table 4. GO categories for modules that comprise cv. Chondrolia Chalkidikis transcriptional regulatory network.**

| **Module ID** | **Number of transcripts** | **GO-ID** | **Term** | **P-Value** |
| --- | --- | --- | --- | --- |
| 1 | 17 | GO:0003871 | 5-methyltetrahydropteroyltriglutamate-homocysteine S-methyltransferase activity | 8.67E-03 |
|  |  | GO:0042085 | 5-methyltetrahydropteroyltri-L-glutamate-dependent methyltransferase activity | 8.67E-03 |
|  |  | GO:0008172 | S-methyltransferase activity | 8.67E-03 |
|  |  | GO:0006809 | nitric oxide biosynthetic process | 8.67E-03 |
|  |  | GO:0046209 | nitric oxide metabolic process | 8.67E-03 |
| 2 | 19 | No GOs are overrepresented | | |
| 3 | 25 | GO:0000287 | magnesium ion binding | 4.56E-03 |
| 4 | 17 | GO:0003755 | peptidyl-prolyl cis-trans isomerase activity | 1.82E-03 |
|  |  | GO:0016859 | cis-trans isomerase activity | 1.82E-03 |
|  |  | GO:0006457 | protein folding | 7.48E-03 |
|  |  | GO:0019776 | Atg8 ligase activity | 7.71E-03 |
|  |  | GO:0005775 | vacuolar lumen | 7.71E-03 |
|  |  | GO:0019779 | APG8 activating enzyme activity | 7.71E-03 |
|  |  | GO:0008641 | small protein activating enzyme activity | 7.71E-03 |
|  |  | GO:0019786 | APG8-specific protease activity | 7.71E-03 |
|  |  | GO:0019783 | small conjugating protein-specific protease activity | 7.71E-03 |
|  |  | GO:0015175 | neutral amino acid transmembrane transporter activity | 7.71E-03 |
|  |  | GO:0015172 | acidic amino acid transmembrane transporter activity | 7.71E-03 |
|  |  | GO:0005275 | amine transmembrane transporter activity | 7.71E-03 |
|  |  | GO:0015171 | amino acid transmembrane transporter activity | 7.71E-03 |
| 5 | 27 | GO:0040007 | growth | 5.06E-03 |
| 6 | 17 | GO:0008026 | ATP-dependent helicase activity | 1.07E-03 |
|  |  | GO:0004386 | helicase activity | 1.07E-03 |
|  |  | GO:0070035 | purine NTP-dependent helicase activity | 1.07E-03 |
|  |  | GO:0044434 | chloroplast part | 4.97E-03 |
|  |  | GO:0044435 | plastid part | 6.53E-03 |
|  |  | GO:0009532 | plastid stroma | 7.48E-03 |
|  |  | GO:0009570 | chloroplast stroma | 7.48E-03 |
|  |  | GO:0030077 | plasma membrane light-harvesting complex | 7.71E-03 |
|  |  | GO:0030076 | light-harvesting complex | 7.71E-03 |
|  |  | GO:0042716 | plasma membrane-derived chromatophore | 7.71E-03 |
|  |  | GO:0009635 | response to herbicide | 7.71E-03 |
|  |  | GO:0009636 | response to toxin | 7.71E-03 |
|  |  | GO:0009772 | photosynthetic electron transport in photosystem II | 7.71E-03 |
|  |  | GO:0003849 | 3-deoxy-7-phosphoheptulonate synthase activity | 7.71E-03 |
|  |  | GO:0009423 | chorismate biosynthetic process | 7.71E-03 |
|  |  | GO:0043650 | dicarboxylic acid biosynthetic process | 7.71E-03 |
|  |  | GO:0033587 | shikimate biosynthetic process | 7.71E-03 |
|  |  | GO:0019632 | shikimate metabolic process | 7.71E-03 |
|  |  | GO:0016887 | ATPase activity | 8.33E-03 |
|  |  | GO:0042623 | ATPase activity, coupled | 8.33E-03 |
| 7 | 35 | GO:0009522 | photosystem I | 3.41E-03 |
|  |  | GO:0051716 | cellular response to stimulus | 6.42E-03 |
|  |  | GO:0005488 | binding | 9.05E-03 |
|  |  | GO:0071310 | cellular response to organic substance | 9.57E-03 |
| 8 | 28 | No GOs are overrepresented | | |
| 9 | 16 | GO:0003735 | structural constituent of ribosome | 6.54E-03 |
|  |  | GO:0009059 | macromolecule biosynthetic process | 8.56E-03 |
|  |  | GO:0034645 | cellular macromolecule biosynthetic process | 8.56E-03 |
|  |  | GO:0010467 | gene expression | 8.81E-03 |
|  |  | GO:0005198 | structural molecule activity | 9.00E-03 |
|  |  | GO:0000022 | mitotic spindle elongation | 9.63E-03 |
|  |  | GO:0000226 | microtubule cytoskeleton organization | 9.63E-03 |
|  |  | GO:0007051 | spindle organization | 9.63E-03 |
|  |  | GO:0051231 | spindle elongation | 9.63E-03 |
|  |  | GO:0007052 | mitotic spindle organization | 9.63E-03 |
| 10 | 23 | GO:0043481 | anthocyanin accumulation in tissues in response to UV light | 6.74E-03 |
|  |  | GO:0043476 | pigment accumulation | 6.74E-03 |
|  |  | GO:0043473 | pigmentation | 6.74E-03 |
|  |  | GO:0043480 | pigment accumulation in tissues | 6.74E-03 |
|  |  | GO:0043478 | pigment accumulation in response to UV light | 6.74E-03 |
|  |  | GO:0043479 | pigment accumulation in tissues in response to UV light | 6.74E-03 |
|  |  | GO:0010329 | auxin efflux transmembrane transporter activity | 6.74E-03 |
|  |  | GO:0015562 | efflux transmembrane transporter activity | 6.74E-03 |
|  |  | GO:0010541 | acropetal auxin transport | 6.74E-03 |
|  |  | GO:0009958 | positive gravitropism | 6.74E-03 |
|  |  | GO:0005221 | intracellular cyclic nucleotide activated cation channel activity | 6.74E-03 |
|  |  | GO:0043855 | cyclic nucleotide-gated ion channel activity | 6.74E-03 |
|  |  | GO:0005217 | intracellular ligand-gated ion channel activity | 6.74E-03 |
|  |  | GO:0006813 | potassium ion transport | 6.74E-03 |
|  |  | GO:0005242 | inward rectifier potassium channel activity | 6.74E-03 |
|  |  | GO:0022834 | ligand-gated channel activity | 6.74E-03 |
|  |  | GO:0005267 | potassium channel activity | 6.74E-03 |
|  |  | GO:0015276 | ligand-gated ion channel activity | 6.74E-03 |
|  |  | GO:0005249 | voltage-gated potassium channel activity | 6.74E-03 |
|  |  | GO:0030551 | cyclic nucleotide binding | 6.74E-03 |
| 11 | 25 | GO:0006725 | cellular aromatic compound metabolic process | 4.52E-03 |
| 12 | 19 | GO:0046686 | response to cadmium ion | 3.17E-04 |
|  |  | GO:0044459 | plasma membrane part | 7.26E-04 |
|  |  | GO:0010038 | response to metal ion | 2.96E-03 |
|  |  | GO:0005886 | plasma membrane | 5.27E-03 |
|  |  | GO:0016192 | vesicle-mediated transport | 6.36E-03 |
|  |  | GO:0012505 | endomembrane system | 7.59E-03 |
|  |  | GO:0010035 | response to inorganic substance | 9.17E-03 |
| 13 | 6 | No GOs are overrepresented | | |
| 14 | 11 | GO:0008756 | o-succinylbenzoate-CoA ligase activity | 2.89E-03 |
|  |  | GO:0042372 | phylloquinone biosynthetic process | 2.89E-03 |
|  |  | GO:0042375 | quinone cofactor metabolic process | 2.89E-03 |
|  |  | GO:0006775 | fat-soluble vitamin metabolic process | 2.89E-03 |
|  |  | GO:0045426 | quinone cofactor biosynthetic process | 2.89E-03 |
|  |  | GO:0042373 | vitamin K metabolic process | 2.89E-03 |
|  |  | GO:0042362 | fat-soluble vitamin biosynthetic process | 2.89E-03 |
|  |  | GO:0042374 | phylloquinone metabolic process | 2.89E-03 |
|  |  | GO:0042371 | vitamin K biosynthetic process | 2.89E-03 |
|  |  | GO:0016877 | ligase activity, forming carbon-sulfur bonds | 5.77E-03 |
|  |  | GO:0016878 | acid-thiol ligase activity | 5.77E-03 |
|  |  | GO:0016405 | CoA-ligase activity | 5.77E-03 |
|  |  | GO:0009108 | coenzyme biosynthetic process | 5.77E-03 |
| 15 | 22 | No GOs are overrepresented | | |
| 16 | 11 | GO:0009509 | chromoplast | 7.70E-03 |
| 17 | 11 | GO:0005198 | structural molecule activity | 3.46E-03 |
|  |  | GO:0032991 | macromolecular complex | 5.46E-03 |
|  |  | GO:0008097 | 5S rRNA binding | 7.71E-03 |
|  |  | GO:0030276 | clathrin binding | 7.71E-03 |
|  |  | GO:0080114 | positive regulation of glycine hydroxymethyltransferase activity | 7.71E-03 |
|  |  | GO:0009064 | glutamine family amino acid metabolic process | 7.71E-03 |
|  |  | GO:0016041 | glutamate synthase (ferredoxin) activity | 7.71E-03 |
|  |  | GO:0016643 | oxidoreductase activity, acting on the CH-NH2 group of donors, iron-sulfur protein as acceptor | 7.71E-03 |
|  |  | GO:0015930 | glutamate synthase activity | 7.71E-03 |
| 18 | 7 | GO:0005730 | nucleolus | 6.68E-03 |
| 19 | 16 | GO:0010016 | shoot morphogenesis | 2.08E-03 |
|  |  | GO:0009888 | tissue development | 3.53E-03 |
|  |  | GO:0022621 | shoot system development | 4.39E-03 |
|  |  | GO:0048367 | shoot development | 4.39E-03 |
|  |  | GO:0009653 | anatomical structure morphogenesis | 9.95E-03 |
| 20 | 20 | GO:0046688 | response to copper ion | 5.78E-03 |
|  |  | GO:0008805 | carbon-monoxide oxygenase activity | 5.78E-03 |
|  |  | GO:0016622 | oxidoreductase activity, acting on the aldehyde or oxo group of donors, cytochrome as acceptor | 5.78E-03 |
|  |  | GO:0006278 | RNA-dependent DNA replication | 5.78E-03 |
|  |  | GO:0003964 | RNA-directed DNA polymerase activity | 5.78E-03 |

The first column shows the module number, the second column shows the number of transcripts that comprise the module. Columns three and four show the GO ID and GO Term respectively while the last column shows the *p-*value of the GO term assignment as calculated in FatiGO tool.
